# A Gas Phase Route to [^18^F]fluoroform with Limited Molar Activity Dilution

**DOI:** 10.1038/s41598-019-50747-3

**Published:** 2019-10-16

**Authors:** Bo Yeun Yang, Sanjay Telu, Mohammad B. Haskali, Cheryl L. Morse, Victor W. Pike

**Affiliations:** Molecular Imaging Branch, National Institute of Mental Health, National Institutes of Health, Building 10, Room B3 C346A, 10 Center Drive, Bethesda, MD 20892-1003 USA

**Keywords:** Synthetic chemistry methodology, Diagnostic markers

## Abstract

Positron emission tomography (PET) is an important imaging modality for biomedical research and drug development. PET requires biochemically selective radiotracers to realize full potential. Fluorine-18 (*t*_1/2_ = 109.8 min) is a major radionuclide for labeling such radiotracers but is only readily available in high activities from cyclotrons as [^18^F]fluoride ion. [^18^F]fluoroform has emerged for labeling tracers in trifluoromethyl groups. Prior methods of [^18^F]fluoroform synthesis used difluoro precursors in solution and led to high dilution with carrier and low molar activity (*A*_m_). We explored a new approach for the synthesis of [^18^F]fluoroform based on the radiosynthesis of [^18^F]fluoromethane from [^18^F]fluoride ion and then cobalt^III^ fluoride mediated gas phase fluorination. We estimate that carrier dilution in this process is limited to about 3-fold and find that moderate to high *A*_m_ values can be achieved. We show that [^18^F]fluoroform so produced is highly versatile for rapidly and efficiently labeling various chemotypes that carry trifluoromethyl groups, thereby expanding prospects for developing new PET radiotracers.

## Introduction

Positron emission tomography (PET) is an increasingly important molecular imaging modality for drug development^1,2^, biomedical research^3^, and medical diagnosis^4–6^. The value of PET for imaging molecular targets in living animal^7^ and human^8^ subjects derives from the development of biochemically specific radiotracers (i.e., radiotracers that are each capable of imaging a single targeted protein, such as a low density neuroreceptor). One of the most useful and widely used radionuclides for labeling such radiotracers is the short-lived positron-emitter, fluorine-18 (*β*^+^  = 97%, *t*_1/2_ = 109.8 min)^9,10^. Nowadays, fluorine-18 can be produced in very high activities (~500 GBq) as aqueous [^18^F]fluoride ion with moderate to high molar activity (*A*_m_; where *A*_m_ is defined^11^ as the ratio of the radioactivity of a compound to its mass at a specified time), typically in the 40–400 GBq/μmol range. Therefore, there has been a surge in the development of methods for the late-stage labeling of PET radiotracers with [^18^F]fluoride ion. However, these methods have been confined mostly to labeling monofluorocarbon (C−F) groups^12,13^.

**Figure 1:**
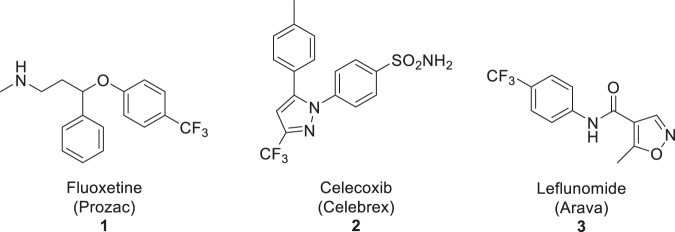
Examples of prominent drugs containing trifluoromethyl groups.

**Figure 2:**
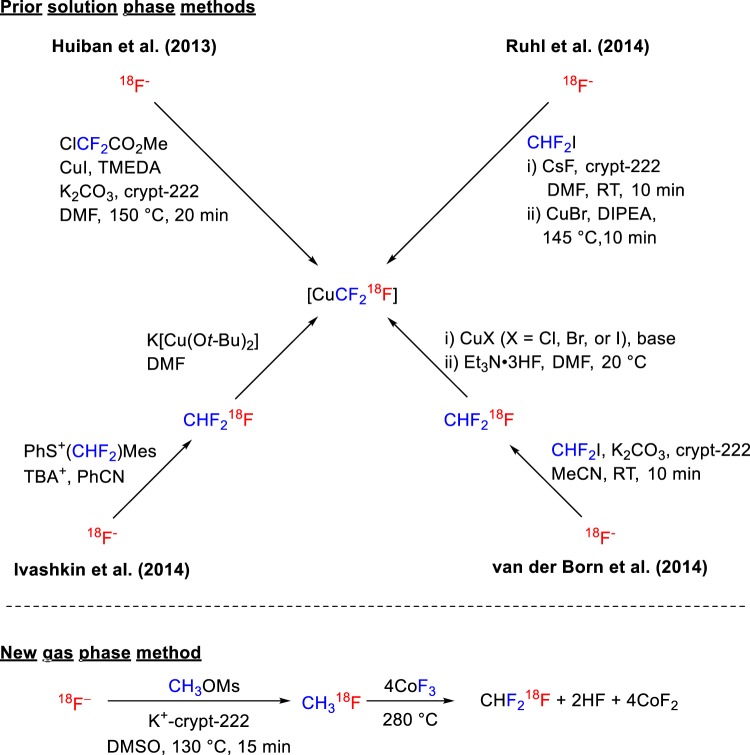
Methods for the radiosynthesis of [^18^F]fluoroform or the derivative [^18^F]CuCF_3_. Prior methods generate [^18^F]fluoroform in solution from a difluoro precursor for use *in situ* or in another solvent. The new method reported here produces [^18^F]fluoroform in the gas phase from [^18^F]fluoromethane.

Substitution of a methyl, chloro, or another substituent in a drug-like molecule with a trifluoromethyl (CF_3_) group can lead to better pharmaceutical properties and improved metabolic stability^14–17^. Consequently, a CF_3_ group regularly appears in many new drugs and drug candidates^18–22^. Prominent examples include fluoxetine (**1**; Prozac), celecoxib (**2**; Celebrex), and leflunomide (**3**; Arava) (Fig. 1). Because of the role of PET in drug development and a frequent requirement to label drugs and new radiotracers with a positron-emitter, academic groups have pursued the development of methods for labeling CF_3_ groups with fluorine-18^23,24^, with the most recent methods being based on generation of [^18^F]CuCF_3_ from [^18^F]fluoride ion either directly or via synthesis of [^18^F]fluoroform (Fig. 2)^25–29^. To date, these solution-phase methods of [^18^F]fluoroform and [^18^F]CuCF_3_ synthesis have delivered at best only very low to moderate *A*_m_ (0.1–32 GBq/µmol), likely due to [^18^F]fluoride ion dilution with carrier fluoride ion originating from the difluoro-precursor [difluorohalomethane, methylchlorodifluoroacetate, or (difluoromethyl)(mesityl)(phenyl)-sulfonium salt] under the reaction conditions (Fig. 2). Generally, however, the molar activities that are needed for radiotracers to be used for PET imaging of low-density protein targets are at the high end of the achievable range or ideally even higher. Here we explored the radiosynthesis of [^18^F]fluoroform according to a different strategy involving initial installation of the fluorine-18 followed by subsequent gas phase difluorination. We find that carrier dilution with this method is limited to about 3-fold. We further show that the [^18^F]fluoroform so produced is useful for preparing a wide range of ^18^F-trifluoromethylated compounds through diverse radiochemical methods^30–32^.

**Figure 3:**
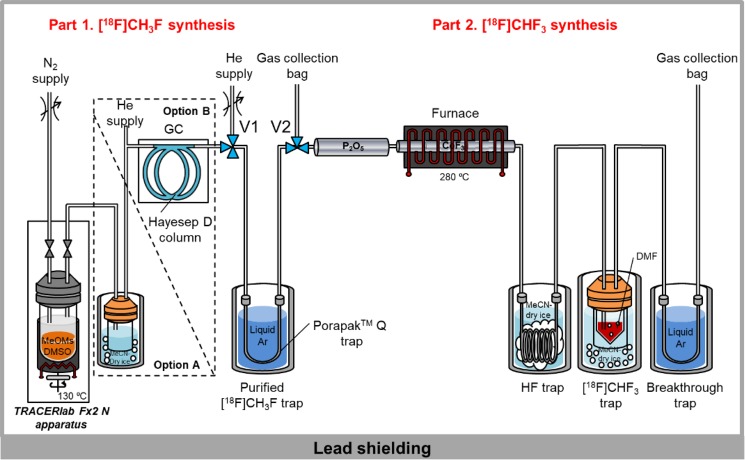
Apparatus for synthesizing [^18^F]fluoroform. Option A was used until it was replaced with Option B for GC purification of [^18^F]fluoromethane. Full technical details on the construction of this apparatus from commercially available components are described in Supplementary Information. In normal operation of the apparatus, the HF and breakthrough traps are not required.

We recently reported a robust and efficient method for the radiosynthesis of [^11^C]fluoroform at very high *A*_m_, based on gas phase fluorination of cyclotron-produced [^11^C]methane with heated cobalt^III^ fluoride (CoF_3_)^33^. We noted that CoF_3_ has also been used to convert fluoromethane into fluoroform. Therefore, to implement our new strategy for the radiosynthesis of [^18^F]fluoroform^34^, we aimed to convert cyclotron-produced [^18^F]fluoride ion into [^18^F]fluoromethane for subsequent difluorination over heated CoF_3_ (Fig. 2). We constructed the apparatus depicted in Fig. 3 for this purpose, except that the indicated gas chromatograph (option B) was introduced in the final stage of our study.

We explored several methods for producing [^18^F]fluoromethane from cyclotron-produced [^18^F]fluoride ion and found that the treatment of methyl mesylate with [^18^F]fluoride ion in DMSO gave an acceptable yield of [^18^F]fluoromethane (46 ± 18%, *n* = 140) after only 15 min. This volatile product (b.p. −78.4 °C) was readily released for collection on a trap of Porapak Q in liquid argon (−186 °C) by purging the reaction mixture with helium at low temperature (35 °C). A trap cooled in dry-ice/MeCN (−41 °C) was used to collect any vaporized non-radioactive organic contaminant before the entrapment of the [^18^F]fluoromethane (Option A). Trapped [^18^F]fluoromethane was released into a helium stream from the warmed Porapak Q trap, and subsequently passed through Sicapent and then over heated CoF_3_. The effluent from the CoF_3_ column was passed through a trap immersed in dry-ice/MeCN to trap any generated acidic species (e.g., potentially HF) and then into either cold ethanol (−72 °C) or DMF to trap the [^18^F]fluoroform (b.p. −82.1 °C). Pilot experiments confirmed the production of [^18^F]fluoroform from this process with the CoF_3_ column operating between 230 and 350 °C.

## Results

### Production of [^18^F]f luoroform

We found that initial conditioning of a newly installed CoF_3_ column by heating it once to 320 °C while sealed under helium resulted in optimal yields of [^18^F]fluoroform in subsequent use at lower temperatures. Conditioning of the column before a run and subsequent regeneration are described in Supplementary Information. The temperature-dependence of the conversion of [^18^F]fluoromethane into [^18^F]fluoroform was investigated with the flow of carrier helium set at 20 mL/min (Supplementary Figs S3 and S4). Only radioactivity trapped in cold (~−72 °C) ethanol was used to calculate the yield (the breakthrough of radioactivity into a subsequent trap was found to be very low: <2%). Moderate yields of [^18^F]fluoroform from [^18^F]fluoromethane were obtained between 280 and 350 °C, with 280 °C appearing optimal (Supplementary Figs S3 and S4).

**Figure 4:**
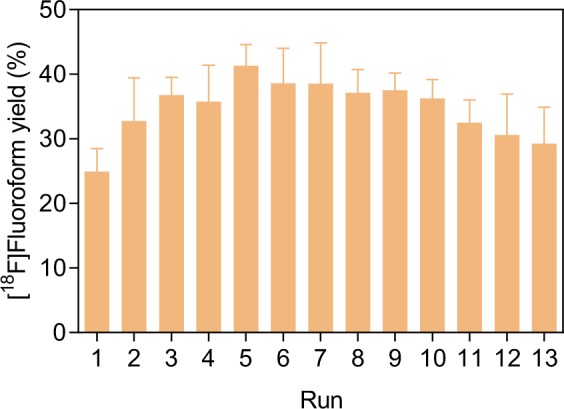
Dependence of [^18^F]fluoroform yield on run number for six different CoF_3_ columns. Data are mean ± SD (*n* = 6).

A single heat-conditioned CoF_3_ column could be used for a series of [^18^F]fluoroform productions (Fig. 4). Yield increased appreciably after the first run and was well maintained over at least 12 subsequent runs. The average yield of [^18^F]fluoroform from [^18^F]fluoromethane was 35 ± 11% (*n* = 77) from six different CoF_3_ columns operated at least a dozen times each. HPLC showed that the only radioactive contaminant was occasionally a very low amount of unchanged [^18^F]fluoromethane (Supplementary Fig. S7). The six CoF_3_ columns produced [^18^F]fluoroform with 98 ± 3% purity (*n* = 77). This good re-usability implies that the CoF_3_ is not rapidly and completely decomposed to CoF_2_ and fluorine at 280 °C. The overall process for producing [^18^F]fluoroform from [^18^F]fluoride ion required 60 minutes from the end of a cyclotron irradiation and was thus much less than one half-life of fluorine-18.

### Investigation of carrier dilution in [^18^F]fluoroform synthesis

**Figure 5:**
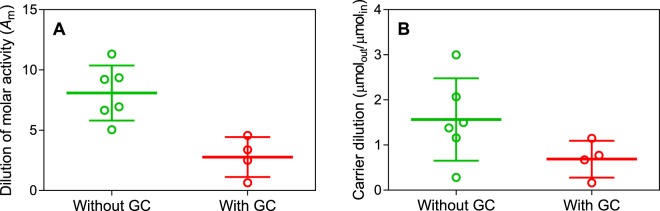
(**A**) Dilution of *A*_m_ for the conversion of [^18^F]fluoromethane into [^18^F]fluoroform without GC purification (Fig. 3, Option A) and with GC purification (Fig. 3, Option B). (**B**) Ratio of carrier amount of fluoromethane entering the CoF_3_ column to that exiting as carrier fluoroform without GC purification and with GC purification.

Of major interest was the *A*_m_ value that could be achieved for the [^18^F]fluoroform that was produced from this method. To estimate *A*_m_, we converted the [^18^F]fluoroform into [^18^F]2,2,2-trifluoro-1,1-diphenylethan-1-ol ([^18^F]**5**) by treatment with benzophenone (**4**) and *t*-BuOK in DMF. [^18^F]**5** was obtained in quantitative yield. The accompanying carrier was measured with a radio-HPLC apparatus having an absorbance detector response at *λ* = 215 nm that was calibrated for the injected mass of **5**. In parallel, we measured the *A*_m_ value of an ^18^F-labeled tracer ([^18^F]*N*-(5-(((2 *S*,4 *S*)-2-methyl-4-(6-fluoropyridin-2-yloxy)piperidin-1-yl)methyl)thiazol-2-yl)acetamide; [^18^F]OGA-1), produced by nucleophilic substitution of an aryl nitro group with [^18^F]fluoride ion^35,36^, in order to estimate the *A*_m_ value of the starting cyclotron-produced [^18^F]fluoride ion. ([^18^F]OGA-1 was being produced in our laboratory for PET imaging of brain *O*-GlcNAcase)^36^. The *A*_m_ value of [^18^F]OGA-1 was taken to be that of the [^18^F]fluoromethane produced from the same batch of [^18^F]fluoride ion i.e., we reasonably assumed that neither non-radioactive precursor (OGA-1 precursor or MeOMs) added appreciable carrier in the labeling reactions. The *A*_m_ of [^18^F]fluoroform was found to be about 8.1−fold lower on average than that of [^18^F]OGA-1 produced from the same stock of [^18^F]fluoride ion when using the apparatus in Fig. 3 with Option A i.e., with no GC purification (Fig. 5A, Supplementary Table S1).

We considered that some of the lower *A*_m_ of [^18^F]fluoroform relative to that of [^18^F]OGA-1 might be due to some generation of fluoromethane through pyrolysis and fluorination of low-level organic impurities in the [^18^F]fluoromethane that reach the CoF_3_ column. To examine this possibility, we installed a small modular gas chromatograph into the hot-cell to purify the [^18^F]fluoromethane before entry into the CoF_3_ column (Fig. 3, option B) and then several times compared the *A*_m_ of [^18^F]**5** produced from the generated [^18^F]fluoroform with that of [^18^F]OGA-1 from the same batch of [^18^F]fluoride ion (Fig. 5A). We found that the dilution of *A*_m_ was reduced on average from 8.1 to 2.8−fold when GC purification of [^18^F]fluoromethane was implemented.

From the *A*_m_ values and measurements of radioactivity entering and leaving the CoF_3_ column, we calculated that in the absence of GC purification the average number of moles of carrier fluoroform produced was 2.01 ± 1.56−fold greater than the number of moles of fluoromethane introduced into the CoF_3_ column. When GC purification was used, this ratio became closer to unity (0.69 ± 0.41−fold) (Fig. 5B, Supplementary Table S4). The latter finding is consistent with our observation that recovery of radioactivity from the CoF_3_ column was 34%, implying that the rest (66%) was retained on the CoF_3_ column. The retained activity was not identified but is clearly not [^18^F]fluoromethane or [^18^F]fluoroform because we had found earlier that no radioactivity adheres to the CoF_3_ column in the conversion of [^11^C]methane into [^11^C]fluoroform^33^.

To explain our observations on carrier dilution and yield, and the radioactivity retained on the CoF_3_ column, we postulate that there is exchange of ^18^F between [^18^F]fluoroform and the co-produced two equivalents of HF (Fig. 2), and that all the radioactive HF adheres to the CoF_3_ column. No radioactivity was ever detected in the depicted HF trap of the apparatus, which is now regarded as redundant. According to our postulate, the yield of [^18^F]fluoroform from [^18^F]fluoromethane at equilibrium is expected to be 33% and the carrier dilution 3-fold, which within likely experimental errors, accords with our observations.

Most of our runs to produce [^18^F]fluoroform were performed at varying periods up to several hours after the end of radionuclide production. To bench-mark comparisons, all estimated *A*_m_ values were decay-corrected to the end of radionuclide production. The maximal molar activity of the [^18^F]fluoride ion available to us was 336 GBq/µmol and on average was 150 ± 73 GBq/µmol (*n* = 10). We found that [^18^F]fluoroform could be produced with an *A*_m_ up to 163 GBq/µmol with an average of 38 ± 35 GBq/µmol, (*n* = 20).

### Trifluoromethylations with [^18^F]fluoroform

**Figure 6:**
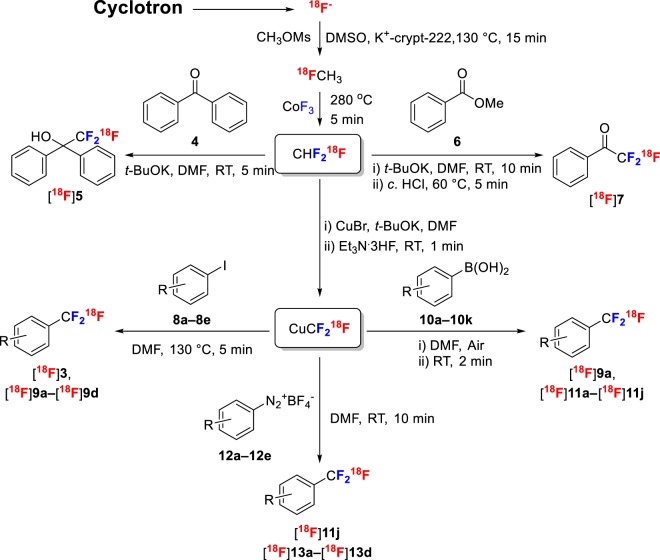
Preparation and use of [^18^F]fluoroform for labeling aryl organic compounds with trifluoromethyl groups. [^18^F]Fluoroform may react directly, as in the topmost examples with an aryl ketone or aryl carboxylic ester, or may be converted rapidly into [^18^F]CuCF_3_ for reaction with, iodoarenes, arylboronic acids, or aryldiazonium salts.

**Figure 7:**
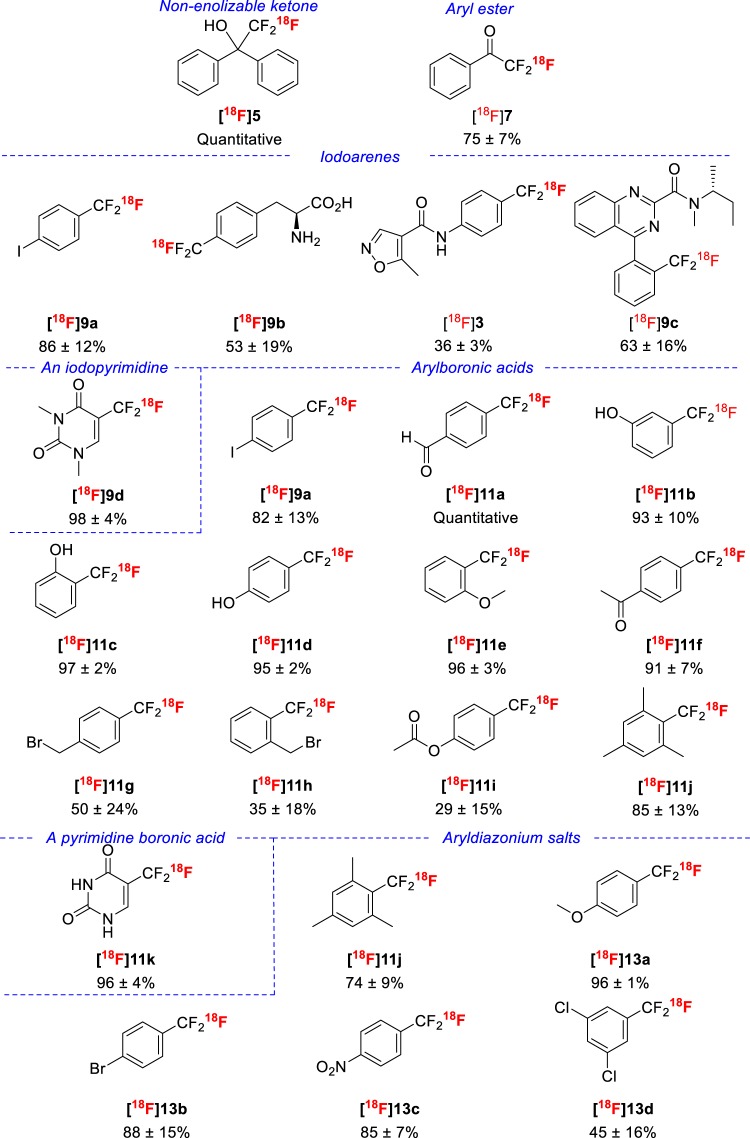
Yields (mean ± SD, *n* = 3) of ^18^F-labeled trifluoromethyl compounds from diverse substrate classes and [^18^F]fluoroform or its copper(I) derivative measured with HPLC. Radiosynthesis methods are summarized in Fig. 6. Blue text indicates the reaction precursor class.

Treatment of benzophenone (**4**) with [^18^F]fluoroform in DMF under basic conditions gave the [^18^F]2,2,2-trifluoro-1,1-diphenylethan-1-ol ([^18^F]**5**) almost quantitatively (Figs 6 and 7), as previously reported^26^. This reaction was useful for molar activity estimations. Treatment of methyl benzoate (**6**) in the presence of *t*-BuOK with [^18^F]fluoroform in DMF at RT followed by treatment with acid gave [^18^F]trifluoroacetylbenzene ([^18^F]**7**) in high yield (75%) as a representative of an entirely new ^18^F-labeled chemotype (Figs 6 and 7).

### Cu(I)-mediated trifluoromethylations with [^18^F]fluoroform

We tested the reactivity of the Cu(I) derivative of the [^18^F]fluorofrom from the new method of radiosynthesis on several model substrates with various methods (Fig. 6). We first confirmed the known reactivity of [^18^F]CuCF_3_ towards iodoarenes^27^. Thus, 1,4-diiodobenzene gave [^18^F]4-trifluoromethyliodobenzene ([^18^F]**9a**), a potentially useful labelling synthon, in high yield (86 ± 12%) (Fig. 7) comparable to that obtained through the same reaction by van der Born *et al*. (73 ± 6%)^29^. This method also gave a previously unknown labeled amino acid [^18^F]**9b** in moderate yield (53 ± 19%). Similarly, an ^18^F-labeled pyrimidine, [^18^F]**9d**, was readily obtained in almost quantitative yield. By use of an iodo precursor, we were able to label the drug leflunomide (**3**) in moderate yield (36 ± 3%), exceeding that reported by Ivashkin *et al*. (18%) for the same reaction^27^. Finally, we demonstrated that we could label a new radioligand for PET imaging of TSPO ([^18^F]**9c**) from an iodo precursor in high non-optimized yield (63 ± 16%).

We also tested the reactivity of [^18^F]CuCF_3_ towards arylboronic acids^27^ with many previously untested examples. Many substituted arylboronic acids gave the corresponding ^18^F-labeled trifluoromethylarenes ([^18^F]**9a**, [^18^F]**11a**−**11j**) in excellent yields when treated with [^18^F]CuCF_3_ for 2 minutes at room temperature. Our results demonstrated the tolerance of this method for CHO, OH, MeO, Ac, Me, and I substituents (Fig. 7). The very high yield of [^18^F]**9a** from this method (82 ± 13%) was very similar to that which we obtained from an iodo precursor, and far exceeded that previously obtained by van der Born *et al*. from the same reaction (4 ± 2%)^29^. For other examples (**11a**, **11d**, **11f**), our yields were very high (>91%) and in accord with those previously reported^29^. The more labile BrCH_2_ and AcO substituents were less well tolerated, giving moderate yields under non-optimized conditions. Nonetheless, these examples ([^18^F]**11 g**–[^18^F]**11i)** show the potential for developing new and useful labeling synthons. The use of a boronic acid precursor gave [^18^F]5-trifluoromethlyuracil ([^18^F]**11k**) in almost quantitative yield.

Finally, the treatment of commercially available ‘wet’ diazonium salts **12a**–**12e** with [^18^F]CuCF_3_ gave [^18^F]**11j**, and [^18^F]**13a**–[^18^F]**13d**, respectively, in good to high yields (Fig. 7). The yield of [^18^F]**11j** (74 ± 9%) was comparable to that from the use of boronic acid as precursor (85 ± 13%). The yields of [^18^F]**13a**–[^18^F]**13c** exceeded 86% and compare well with the yields of these labeled compounds from the use of arylboronic acids or aryl iodides as precursors^28^. This new method therefore appeared highly effective for the simple one-pot conversion of arylamines into [^18^F]trifluoromethylarenes.

## Discussion

[^18^F]f luoroform was readily produced in useful yield and with limited carrier dilution from cyclotron-produced [^18^F]fluoride ion by passing [^18^F]fluoromethane over heated CoF_3_. Because our results indicate that carrier dilution is limited to about 3-fold in this new method of [^18^F]fluoroform production, we expect that [^18^F]fluoroform of even higher molar activity could be produced from sources of [^18^F]fluoride ion of higher molar activity in a directly proportional manner. It would therefore be interesting to see how this method performs with [^18^F]fluoride ion of much higher *A*_m,_ as is typically available in some laboratories. A difluorocarbene intermediate has been construed to occur in other methods of [^18^F]fluoroform or [^18^F]CuCF_3_ synthesis from difluoro precursors and to be a major source of carrier dilution. Prior methods of [^18^F]fluoroform/[^18^F]CuCF_3_ synthesis may be capable of delivering higher molar activities than so far reported by using much higher levels of starting radioactivity and by limiting the amount of difluorocarbene formation. The radiochemical pathway in our new method for producing [^18^F]fluoroform clearly avoids any possibility for carrier dilution from difluorocarbene formation. The radiosynthesis apparatus is considered amenable to automation and remote control to ensure radiation protection for personnel. With this method, the labeling of PET radiotracers at a trifluoromethyl group with usefully high *A*_m_ becomes possible. Although the overall yield of [^18^F]fluoroform appears modest, the speed, broad scope, and generally high efficiency seen in the many examples of labeling reactions augurs well for useful application of this new method. This is especially so given that very high activities of [^18^F]fluoride ion can be produced on modern cyclotrons (>400 GBq). With this method, we now envisage access to an enhanced range of useful and exciting radiotracers for PET based on adapting the known richly diverse chemistry of fluoroform^37–40^ and its derivatives^41–51^ for unprecedented ^18^F-labeling at trifluoromethyl groups. These radiotracers may include chemotypes never previously labeled with fluorine-18.

## Materials and Methods

Sources of materials are detailed in Supplementary Information.

### Synthesis of [^18^F]fluoroform

The apparatus depicted in Fig. 3 was constructed, set-up, and operated as detailed in Supplementary Information. Transfers of radioactivity through the apparatus were monitored with PIN-diode detectors. [^18^F]Fluoride ion was produced on a cyclotron (PETtrace; GE) according to the ^18^O(p,n)^18^F reaction by irradiating ^18^O-enriched water (3 mL, 98 atom %) with a beam of protons (16.5 MeV; 50 µA) for at least 45 min. [^18^F]Fluoromethane was synthesized within a fully automated apparatus (TRACERlab^TM^ FX2N; GE). Thus, [^18^F]fluoride ion (1.9–14.8 GBq) in [^18^O]water (200–400 µL) and a solution (100 µL) containing K_2_CO_3_ (10 µmol) plus crypt-222 (20 µmol) were loaded into a glass vial. MeCN (2 mL) was added and the solvent was azeotropically removed at 88 °C under a stream of nitrogen gas that was vented to vacuum. This step was repeated two more times. A solution of MeOMs (0.1 mmol, 8.5 µL) in anhydrous DMSO (1 mL) was then added to the dried [^18^F]F^–^K^+^- crypt-222 complex, sealed, and heated at 130 °C for 15 min. The reaction vial was then cooled to 35 °C. [^18^F]Fluoromethane (b.p. −78.4 °C) was flushed out of the vial with nitrogen gas (20 mL/min) and into Porapak Q (80–100 mesh; 1 g) contained in a first U-shaped stainless-steel tube (0.069 in i.d.) cooled with liquid argon (−186 °C). The transfer generally required 5 min. The sealed trap was then removed from the cooling bath and measured for radioactivity at RT (20–26 °C) with a dose calibrator. The [^18^F]fluoromethane was then released into a stream of helium gas (20 mL/min) from the Porapak Q trap through Sicapent (phosphorus pentoxide) and then through a heated column (280 °C) of CoF_3_ (19 g) for a period of 7 to10 min. The generated [^18^F]fluoroform was passed through a trap cooled in dry-ice/MeCN (−41 °C) and finally into a glass V-vial containing DMF (0.6–0.8 mL) that was cooled also in a dry-ice/MeCN bath. A second U-shaped stainless-steel tube containing Porapak Q (80–100 mesh) was connected to the outlet of the V-shaped glass product vial to retain any breakthrough of radioactive material for measurement.

### Trifluoromethylation reactions

#### Synthesis of [^18^F]2,2,2-trifluoro-1,1-diphenylethan-1-ol ([^18^F]5)

Benzophenone (**4**; 55 µmol, 9 mg) was put into a 1-mL glass vial with a solution of *t*-BuOK (0.3 M) in DMF (150 µL) and capped with a septum seal. [^18^F]f luoroform in DMF (100–300 µL) was added to the vial, and the mixture was left to react at RT for 5 min.

#### Synthesis of [^18^F]trifluoroacetylbenzene ([^18^F]7)

2-Methyl benzoate (**6**; 50 µmol, 7 mg) was put into a 1-mL glass vial with *t*-BuOK DMF (0.3 M, 50 µL) and capped with a septum seal. [^18^F]f luoroform in DMF (100–300 µL) was added, and the mixture was left at RT for 10 min. Hydrochloric acid (37%, 0.1 mL) was added and heated at 60 °C for 5 min. The mixture was quenched with aq. 0.1% TFA/MeCN (1:1, v/v) solution and filtered through a PTFE syringe filter (0.2 µm pore size).

#### [^18^F]CuCF_3_ synthesis

CuBr (5 µmol, 0.7 mg) was added to 1-mL glass vial and moved to a glove box (dry nitrogen atmosphere). *t*-BuOK in DMF (0.3 M, 50 µL) was added to the vial, which was then septum-sealed and removed from the glove box. [^18^F]f luoroform in DMF (50–300 µL) was added to the vial, mixed, and left at RT for 1 min. A solution of Et_3_N**·**3HF in DMF (1.64% v/v, 5 mL) was then added. The mixture was mixed thoroughly and allowed to stay at RT for another minute before use in labeling reactions.

#### Syntheses of [^18^F]trifluoromethylarenes from aryl iodides and [^18^F]CuCF_3_

Aryl iodide precursor (100 µmol) in DMF (150 µL) was added to a prepared vial of [^18^F]CuCF_3_ and shaken vigorously. The mixture was heated at 130 °C for 5 min, quenched with aq. 0.1% TFA/MeCN (1:1, v/v) solution, and finally filtered through a PTFE syringe filter (0.2 µm pore size).

#### Syntheses of [^18^F]trifluoromethylarenes from arylboronic acids and [^18^F]CuCF_3_

Arylboronic acid precursor (50 µmol) in DMF (100 µL) was added to a prepared vial of [^18^F]CuCF_3_ and shaken vigorously. Air was passed from 10-mL syringe into the vial., and out through a vent needle. The reaction mixture was left at RT for 2 min, quenched with aq. 0.1% TFA/MeCN (1:1, v/v) solution, and finally filtered through a PTFE syringe filter (0.2 µm pore size).

#### Syntheses of [^18^F]trifluoromethylarenes from aryldiazonium salts and [^18^F]CuCF_3_

Aryldiazonium salt precursor (50 µmol) in DMF (100 µL) was added to a prepared vial of [^18^F]CuCF_3_ and shaken vigorously. The reaction mixture was left at RT for 10 min, quenched with aq. 0.1% TFA/MeCN (1:1, v/v) solution, and finally filtered through a PTFE syringe filter (0.2 µm pore size).

#### Radiochemical analysis

Methods are described in Supplementary Information.

### Statistical analyses

Two-tailed unpaired Student’s *t*-test (α = 0.05) were used for comparisons between two *A*_m_ values (GBq/µmol). Grouped data are presented as mean ± SD. All statistical data were calculated using Prism software v5.02 (GraphPad, San Diego, CA, USA).

## Supplementary information

Supplementary Information

## Data Availability

All data generated or analysed during this study are included in this published article (and its Supplementary Information Files).
