# Supplementary material for: A Gas Phase Route to [18F]fluoroform with Limited Molar Activity Dilution
Source: Sci Rep. 2019 Oct 16;9:14835. doi: 10.1038/s41598-019-50747-3 (PMC6795885; doi:10.1038/s41598-019-50747-3)
Supplement: Supplementary file 1 — Supplementary Information [file 41598_2019_50747_MOESM1_ESM.pdf]

ELECTRONIC SUPPLEMENTARY INFORMATION

A Gas Phase Route to [^{18}F]Fluoroform with Limited Molar Activity Dilution

Bo Yeun Yang, Sanjay Telu, Mohammad B. Haskali, Cheryl L. Morse, and Victor W. Pike*

Molecular Imaging Branch, National Institute of Mental Health, National Institutes of Health, Building 10, Room B3 C346A 10 Center Drive, Bethesda, MD, 20892-1003, USA

***Corresponding author:** Victor. W. Pike, Ph.D., Molecular Imaging Branch, National Institute of Mental Health, National Institutes of Health, Building 10, Room B3 C346A, 10 Centre Drive, Bethesda, MD, 20892-1003, USA. pikev@mail.nih.gov

CONTENTS

	Page
MATERIALS AND GENERAL METHODS	S3
SYNTHESES	S3
N-(4-Iodophenyl)-5-methylisoxazole-4-carboxamide.	S3
2,4,6-Trimethylbenzenediazonium tetrafluoroborate	S4
RADIOCHEMISTRY	S4
Description of traps and columns incorporated into the radiosythesis apparatus	S4
[^{18}F]Fluoromethane PoraPak Q trap	S4
Sicapent column	S4
'HF trap'	S4
Cobalt(III) trifluoride column	S4
Breakthrough trap	S5
Radioactive product collection trap	S5
[^{18}F]Fluoromethane Synthesis	S5
[^{18}F]Fluoroform Synthesis	S6
Calculation of [^{18}F]fluoroform yield.	S6
Dependence of [^{18}F]fluoroform yield on amount of CoF_3.	S6
Dependence of [^{18}F]fluoroform purity on CoF_3 column temperature.	S7
Radiochemical Data Analysis	S7
GC-MS analysis of [^{18}F]fluoroform	S7
HPLC analyses of radioactive products - general aspects	S8
[^{18}F]Fluoromethane and [^{18}F]fluoroform analysis	S8
[^{18}F]2,2,2-Trifluoro-1,1-diphenylethan-1-ol ([^{18}F]**5**) analysis	S10
[^{18}F]4-Trifluoroacetylbenzene ([^{18}F]**7**) analysis	S10
Analyses of [^{18}F]trifluoromethylarenes produced from aryl iodides.	S11
Analyses of [^{18}F]trifluoromethylarenes produced from arylboronic acids.	S13
Analyses of [^{18}F]trifluoromethylarenes produced from aryldiazonium salts.	S19
Determination of Molar Activity (A_m) of [^{18}F]**5**	S21
Comparison of the Molar activity of [^{18}F]**5** with that of a Radiotracer, [^{18}F]OGA-1, Produced by S_NAr from the Same Source of [^{18}F]Fluoride ion	S22
Comparison of the Molar Activity of [^{18}F]**5** with that of [^{18}F]**9a** Produced from the Same Source of [^{18}F]Fluoride Ion	S22
Calculation of Dilution of A_m (for Fig. 5A)	S23
Calculation of Carrier Dilutions (for Fig. 5B)	S23
References	S24

MATERIALS AND GENERAL METHODS

Stainless steel seamless tubes (0.375” O.D; 0.035” wall thickness; 20 ft length.; 0.125” O.D; 0.028” wall thickness; 20 ft length; and 0.0625” O.D.; 0.014” wall thickness; 6 m length) were purchased from Swagelok (Solon, OH) and cut to length as needed. PoraPak Q (80−100 mesh; Supelco; Bellefonte, PA) and quartz wool (Grace; Columbia, MD) were used to pack stainless tubes. Stainless steel fittings (3/8” O.D. to 1/8” O.D. reducing union, nut and ferrule set; 3/8” nut and ferrule set) were purchased from Swagelok. Polytetrafluoroethylene (PTFE; Teflon) tubing (0.062” O.D, 0.018−0.022 I.D; 0.125” O.D, 0.058−0.060 I.D) and stainless frits (0.2 µm pore × 1.00 mm thick) were purchased from VICI (Houston, TX). UHP carrier grade helium gas (Roberts Oxygen; Gaithersburg, MD) was used as carrier gas in the radiochemistry. Mass flow controller (0−100 SCCM; 5M, 5IN, Gas: He), power adapter (24 V DC, 1 A), cable (8 pin mini DIN 25’ double-ended), and local setpoint module (w/DC-62 cable) were all purchased from Alicat Scientific, Inc. (Tuscon, AZ). Vial heads with 4-connectors for 1/4-28” UNF were purchased from Eckert & Ziegler GmbH (Berlin, Germany). A high-performance liquid chromatography (HPLC) apparatus (System Gold; Beckman Coulter, Fullerton, CA) was used with a Luna C18(2) column (10 µm, 100 Å, 4.6 × 250 mm; Phenomenex, Torrance, CA). Velos Pro Liquid Chromatography-Mass Spectroscopy (LC-MS) (Thermo Scientific, San Jose, CA) was used with a Luna C18 column (3 µm, 2.0 × 50 mm; Phenomenex). Some non-radioactive products were analyzed with GC-MS on a Polaris Q instrument (Thermo Fisher Scientific; Waltham, MA) using a Restek Rtx-5MS column (0.25 mm I.D. × 30 m length). A TRACERlab FX2 N apparatus (GE Healthcare, Chicago, IL) was used for automated radiosynthesis of [^{18}F]fluoromethane.

Cobalt(III) fluoride (CoF_3) was purchased from Sigma-Aldrich (St. Louis, MO). Anhydrous DMF (extra dry over molecular sieve; Acroseal) was purchased from Acros Organics (Geel, Belgium). 2,4,6-Trimethylbenzenediazonium tetrafluoroborate was prepared as previously described.[1] Iodo precursor (8d) for the labelling of the TSPO ligand [^{18}F]**9c** and the TSPO ligand **9c** as a standard for HPLC analysis were prepared and characterized by Dr. Fabrice Siméon (NIMH, NIH) as being > 95% pure. Other chemicals were purchased from Sigma-Aldrich (St. Louis, MO), AstaTech, Inc. (Bristol, PA), or Oakwood Chemicals (Estill, SC). Fluorine-18 radioactivity was measured with a calibrated dose calibrator (Biodex Medical Systems; Shirley, NY).

SYNTHESES

***N*-(4-Iodophenyl)-5-methylisoxazole-4-carboxamide.** A solution of 4-iodoaniline (1.4 g, 6.8 mmol) plus Et_3N (2 mL) in CH_2Cl_2 (15 mL) was added dropwise to a solution of 5-methylisoxazole-4-carbonyl chloride (1.0 g, 6.8 mmol) in CH_2Cl_2(5 mL) at 0 °C , while maintaining the temperature below 5 °C. The mixture was stirred at 0 °C for 1 h and then warmed to ambient temperature and stirred overnight. The mixture was then washed with sat. aqueous $NaHCO_3$, dried over $MgSO_4$, filtered, and concentrated to an oil. The crude

product was purified with chromatography on silica gel using 20−35% ethyl acetate in hexanes as eluent. The title compound was isolated as a white solid (0.79 g). ^{1}H NMR (CD_3CN) δ 8.67 (s, 1H), 8.49 (bs, 1H), 7.68 (d, J = 8.8 Hz, 2H), 7.49 (d, J = 8.8 *Hz*, 2H), 2.68 (s, 3H). $^{13}C\{^{1}H\}$NMR (CD_3CN) δ 174.7, 160.6, 149.4, 139.5, 138.8, 123.3, 113.0, 87.8, 12.8. HRMS (ESI) *m/z*: $[M + H]^+$ calcd for $C_{11}H_9IN_2O_2$, 328.9709; found, 328.9790.

2,4,6-Trimethylbenzenediazonium tetrafluoroborate. A solution of 2,4,6-trimethylaniline (1.35 g, 10 mmol) in CH_2Cl_2 (20 mL) was added dropwise into $BF_3{\cdot}Et_2O$ (1.85 mL, 15 mmol) at – 15°C. A solution of *t*-BuONO (1.4 mL, 12 mmol) in CH_2Cl_2 (10 mL) was slowly added to the reaction mixture while the temperature was maintained at – 15 °C. The reaction mixture was stirred for a further 20 min and then diluted with pentane (20 mL). The yellow precipitate was filtered off, washed with ice-cold diethyl ether, and dried under vacuum. The title compound was isolated as a pale yellow solid (2.25 g). ^{1}H NMR (CD_3CN) δ 7.41 (s, 2H), 2.65 (s, 6H), 2.51 (s, 3H). $^{19}F\{^{1}H\}$NMR (CD_3CN) δ −151.65, −151.70. $^{13}C\{^{1}H\}$NMR (CD_3CN) δ 156.6, 145.9, 132.4, 111.5, 23.0, 19.0. LC-MS (ESI) *m/z*: $[M + CH_3OH]^+$ calcd for $C_{10}H_{15}N_2O$, 147.0917; found, 179.0.

RADIOCHEMISTRY

Description of traps and columns incorporated into the radiosythesis apparatus

The following are descriptions of traps and colums used in the [^{18}F]fluoroform synthesis apparatus (main text, **Fig. 3**).

[^{18}F]Fluoromethane PoraPak Q trap. Stainless-steel tube (0.069" I.D.; 0.125" O.D.; 10" length) was dried completely in an oven to eliminate moisture and then bent to a U–shape and packed with PoraPak Q (1 g) that was held in place with quartz wool at each end.

Sicapent column. Stainless-steel tube (0.305" I.D.; 0.375” O.D.; 12" length) was dried completely in an oven to eliminate moisture and packed with Sicapent (P_2O_5) that was held in place with quartz wool at each end.

'HF trap'. An empty stainless-steel tube (0.069" I.D.; 0.125" O.D.; 15" length) was coiled and connected to the apparatus. When used to trap any HF, the coil was placed inside a Dewar flask filled with dry ice and acetonitrile (–41 °C).

Cobalt(III) trifluoride column. A stainless-steel tube (0.305" I.D.; 0.375" O.D.; 14" length) was dried completely in an oven (106 °C) to eliminate moisture and then moved to a glove box. Under a dry nitrogen atmosphere, the column was packed with CoF_3 (19 g) that was held in place with glass wool at each end and then closed with stainless-steel nut fittings (Swagelok). The column was taken out of the glove box, and the nuts were removed just before installation. The column was connected to the apparatus immediately after the Sicapent column using an empty stainless tube (0.0345” I.D.; 0.0625” O.D.; 10” length) with

Swagelok fittings. The column was flushed with helium. The CoF_3 column was sealed off while still filled with helium gas and conditioned at first at 320 °C for 30 min. After every radiosynthesis, the CoF_3 column was regenerated by closing the ends of the column, cooling the helium-filled column to between 28 and 30 °C with external compressed air, and then re-heating the column to 280 °C while under continuous purge with helium (20 mL/min) for 20 h.

Breakthrough trap. Stainless steel tube (0.069" I.D.; 0.125" O.D.; 10" length) was dried completely in an oven to eliminate moisture and packed with PoraPak Q (1 g) that was held in place with glass wool at each end.

Radioactive product collection traps. Radioactive products from the [^{18}F]fluoroform synthesis apparatus were collected from the helium gas stream in ethanol (5 mL) that was cooled with a hexane-liquid nitrogen bath (~ –94 °C) or in DMF (1 mL) that was cooled with an acetonitrile-dry ice bath (~ –41 °C). These traps collected virtually all the [^{18}F]fluoroform (b.p. –82.1 °C), as shown by very low (< 2%) breakthrough of radioactivity into the breakthrough trap. These traps were also expected to retain most of any possible radioactive contaminants, such as [^{18}F]fluoromethane (b.p. –78.4 °C) or [^{18}F]difluoromethane (b.p. –52 °C).

[^{18}F]Fluoromethane synthesis

Dry [^{18}F]fluoride ion-K^+-crypt-222 reagent was prepared as described in the main text. A solution of methyl methanesulfonate (MeOMs; b.p. 202−203 °C; 8.5 µL, 0.1 mmol) in anhydrous DMSO (1 mL) was added to the dry septum-sealed vial at 60 °C. The reaction mixture was heated at 130 °C for 15 min and then cooled to 35 °C. The generated [^{18}F]fluoromethane was flushed out of the vial with nitrogen gas at 20 mL/min and into a PoraPak Q trap cooled in liquid argon. Radioactivity in the trap was measured with a calibrated ionization chamber to calculate the yield of [^{18}F]fluoromethane from [^{18}F]fluoride ion. Experiments showed little sensitivity of yield to reaction temperature between 120 and 160 °C (n = 1) (**Fig. S1**). The [^{18}F]fluoromethane was released as needed by warming the trap while it was purged with helium (typically at 20 mL/min).

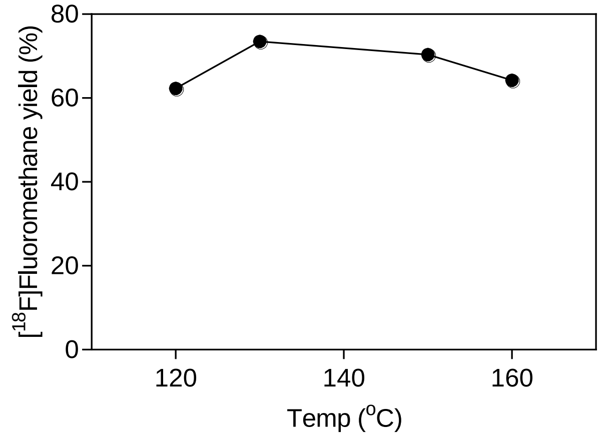

Figure S1. Temperature dependence of [^{18}F]fluoromethane yield from [^{18}F]fluoride ion (n = 1).

[^{18}F]Fluoroform synthesis

Calculation of [^{18}F]fluoroform yield. Starting [^{18}F]fluoromethane and product [^{18}F]fluoroform were trapped respectively in a liquid argon cooled PoraPak Q trap (– 186 °C) and a DMF-dry-ice trap (–41 °C). Both were measured with a calibrated ionization chamber. The percentage recovery of radioactivity was calculated by dividing product activity (MBq) by [^{18}F]fluoromethane activity (MBq) (with decay correction) and multiplying by 100. The purity of [^{18}F]fluoroform in the collected product was determined with HPLC. Multiplication of the radioactivity recovery by the fractional purity gave the percentage yield of [^{18}F]fluoroform from [^{18}F]fluoromethane.

Dependence of [^{18}F]fluoroform yield on amount of CoF_3. [^{18}F]Fluoroform synthesis was performed with a column of either 5 g or 19 g of CoF_3. The highest yield of 52.6% was obtained with 19 g of CoF_3 at 280 °C. [^{18}F]Fluoroform yield from a 5 g CoF_3 column at 280 °C was far lower (15.2%). Both columns gave much lower yield at 230 °C (5 g, 2.68%; 19 g, 23.1%) (**Fig.S2**). No product was obtained at RT.

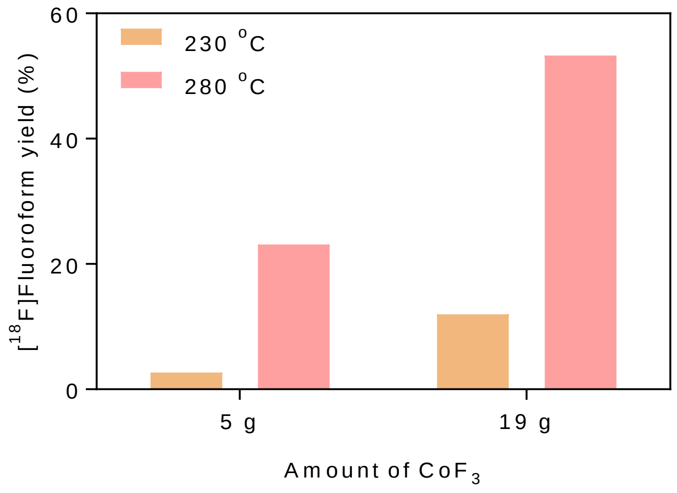

Figure S2. [^{18}F]Fluoroform yields from [^{18}F]fluoromethane conversion with a 5 or 19 g column of CoF_3 at 230 or 280 °C (n = 1).

Dependence of [^{18}F]fluoroform purity on CoF_3 column temperature. The dependence of the purity of collected [^{18}F]fluoroform on CoF_3 column temperature was investigated and found to be quantitative at 280 °C or higher (n = 2) (**Fig. S3**).

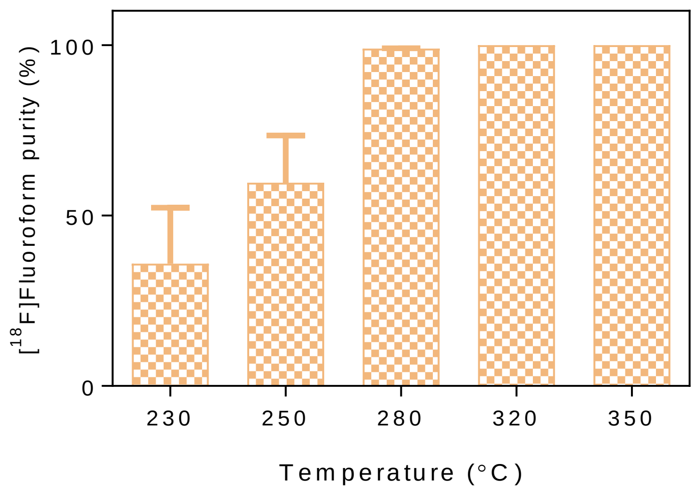

Figure S3. Collected [^{18}F]fluoroform purity versus temperature of CoF_3 column as determined with radio-HPLC.

Dependence of [^{18}F]fluoroform yield on CoF_3 column temperature. The dependence of yield of collected [^{18}F]fluoroform on CoF_3 column temperature was investigated and found to be optimal at 280 °C (52%; n = 2) (**Fig. S4**).

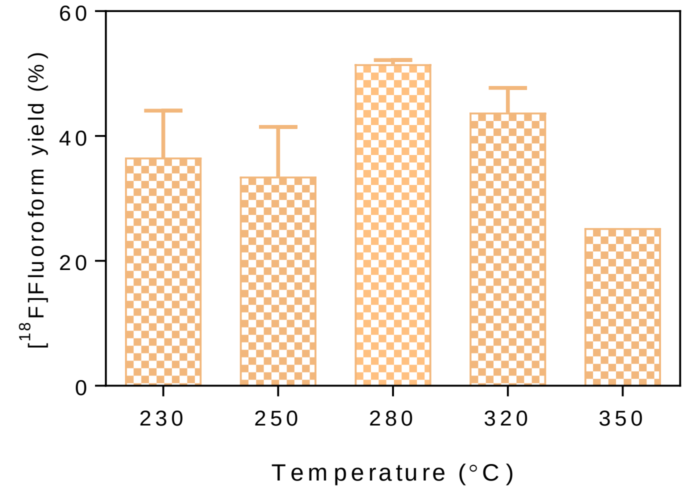

Figure S4. [^{18}F]Fluoroform yield from [^{18}F]fluoromethane versus temperature of CoF_3 column, as determined with radio-HPLC.

Radiochemical Data Analysis

GC-MS analysis of [^{18}F]fluoroform. [^{18}F]Fluoroform was identified through GC-MS of associated carrier. After entrapment of [^{18}F]fluoroform in cold DMF, (~ –41 °C) a sample was added to a septum-sealed vial (1.5 mL; 12 × 32 screw cap; Agilent Technologies; Santa Clara, CA) and sonicated for 10 s. The headspace was sampled with a Hamilton syringe and a portion (2 μL) was injected onto GC-MS for analysis at 100 °C with a helium flow of 1 mL/min. The acquisition was set to electron spray (EI) mode with ion source set

at 200 °C for 5 min. Mass was recorded over the range of 50−150 amu. Ions at *m/z* 69 ($[CF_3]^+$) and 51 ($[CHF_2]^+$) were the only ions observed in accord with the mass spectrum of reference fluoroform (**Fig. S5**).

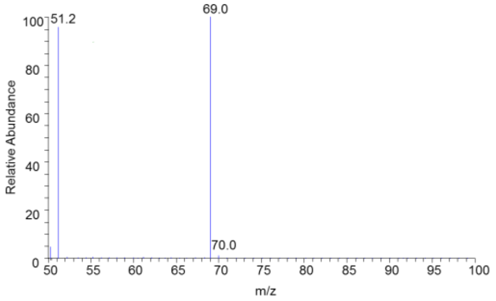

Figure S5. GC-MS chromatogram for fluoroform. $[CF_3]^+$ *m/z* = 69, $[CHF_2]^+$ = 51.

HPLC analyses of radioactive products - general aspects. All radioactive products were analyzed with HPLC on a Luna C18 column (10 µm, 250 × 4.6 mm) eluted at 2 mL/min with the mobile phases later specified for each analyte. Eluates were measured for radioactivity with a scintillation detector and for absorbance at 215 or 254 nm under continuous flow. All products were identified by comparison of their retention times with those of authentic references. For the estimation of radiochemical yields, all radioactive peaks were decay-corrected. Peaks for unchanged [^{18}F]fluoromethane were not included in product yield determinations for radiotrifluoromethylated products. Recovery of analyte radioactivity was checked periodically by collecting all peaks from an analysis for separate measuremnt of radioactvity. No appreciable losses of radioactivity during analyses were observed.

[^{18}F]Fluoromethane and [^{18}F]fluoroform analysis. [^{18}F]Fluoromethane (**Fig. S6**) and [^{18}F]fluoroform (**Fig. S7**) in DMF or methanol were analyzed immediately after collection with a mixture of 0.1% TFA water (A) and MeCN (B) as mobile phase, with B set initially at 55% for 1 min, and then linearly increased to 80% over 15 min.

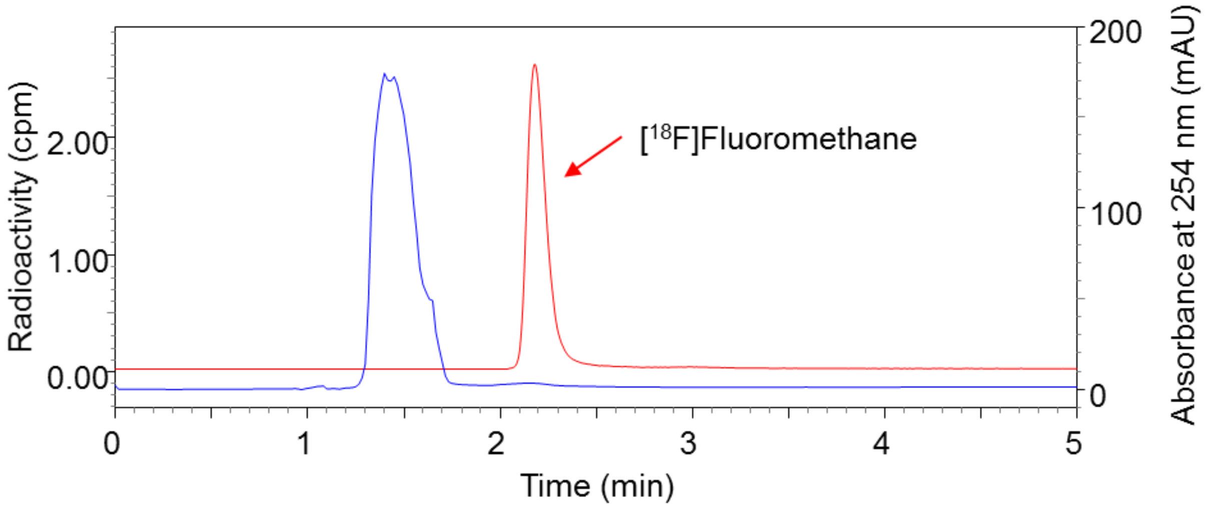

Figure S6. HPLC analysis of [^{18}F]fluoromethane.

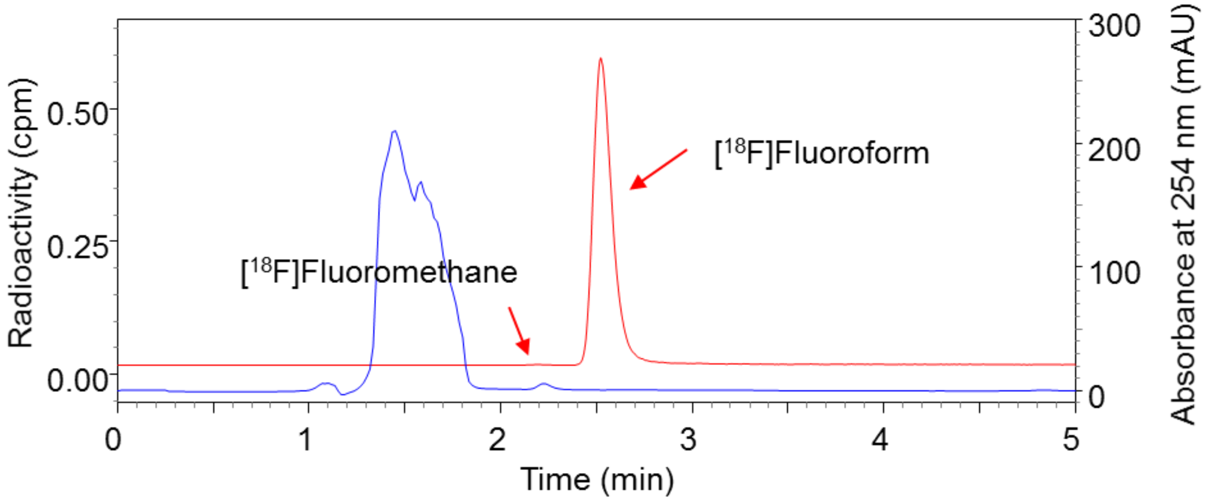

Figure S7. HPLC analysis of [^{18}F]fluoroform.

[¹⁸F]2,2,2-Trifluoro-1,1-diphenylethan-1-ol ([¹⁸F]5) analysis. Crude [^{18}F]**5** was analyzed with a mixture of water (A) and MeCN (B) as mobile phase, with B set initially at 45% for 1 min, and then increased linearly to 80% over 20 min (**Fig. S8**).

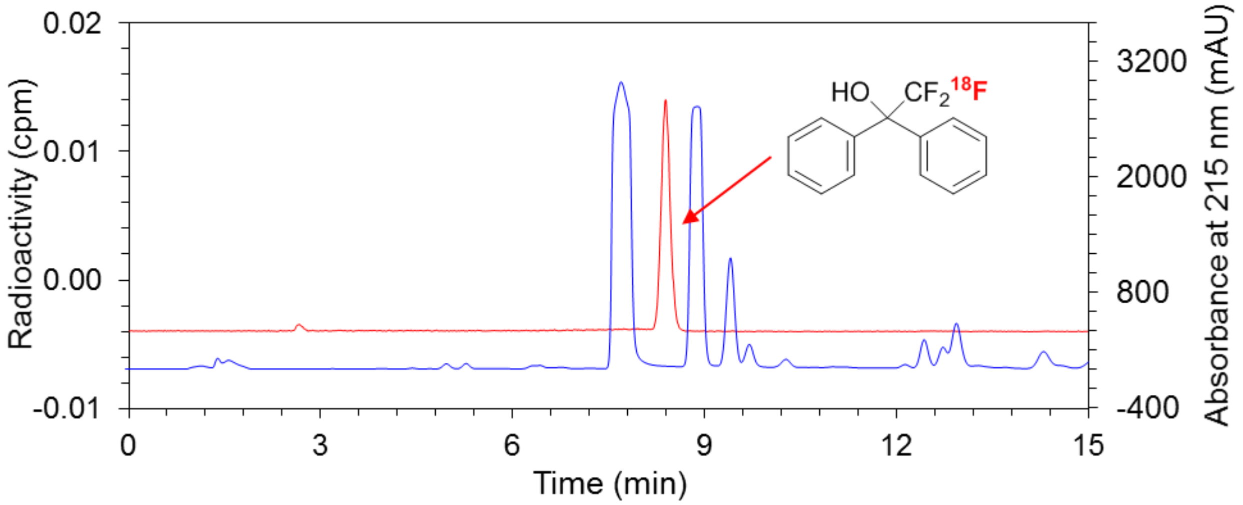

Figure S8. HPLC analysis of crude [^{18}F]2,2,2-trifluoro-1,1-diphenylethan-1-ol ([^{18}F]**5**).

[^{18}F] Trifluoroacetylbenzene ([^{18}F]7) analysis. Crude [^{18}F]**7** was analyzed with a mixture of water (A) and MeCN (B) as mobile phase, with B set initially at 10% for 1 min, and then linearly increased to 80% over 20 min (**Fig. S9**).

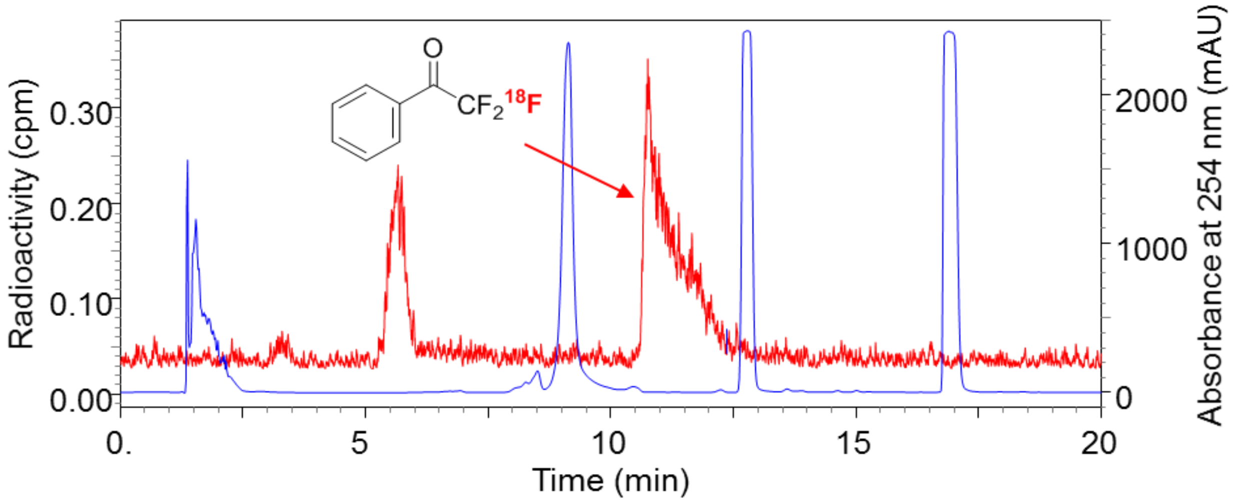

Figure S9. HPLC chromatogram for crude [^{18}F]trifluoroacetybenzene ([^{18}F]**7**).

Analyses of [^{18}F]trifluoromethylarenes produced from aryl iodides.

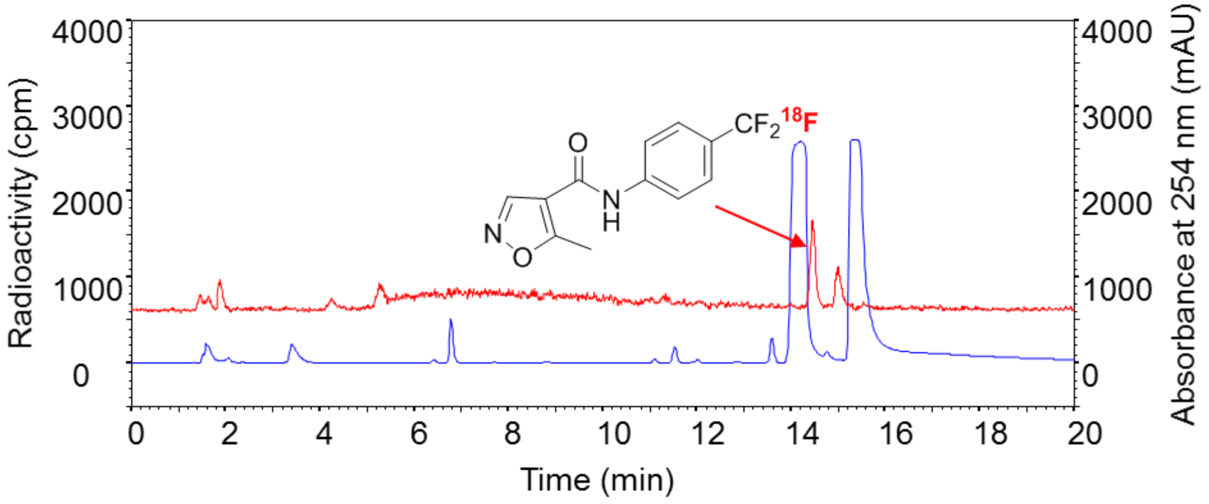

Figure S10. HPLC analysis of crude [^{18}F]5-methyl-*N*-(4-(trifluoromethyl)phenyl)isoxazole-4-carboxamide (Leflunomide) ([^{18}F]**3**) with a mixture of 0.1% TFA water (A) and MeCN (B) as mobile phase, with B initially set at 5% for 1 min, and then increased linarly to 90% over 10 min

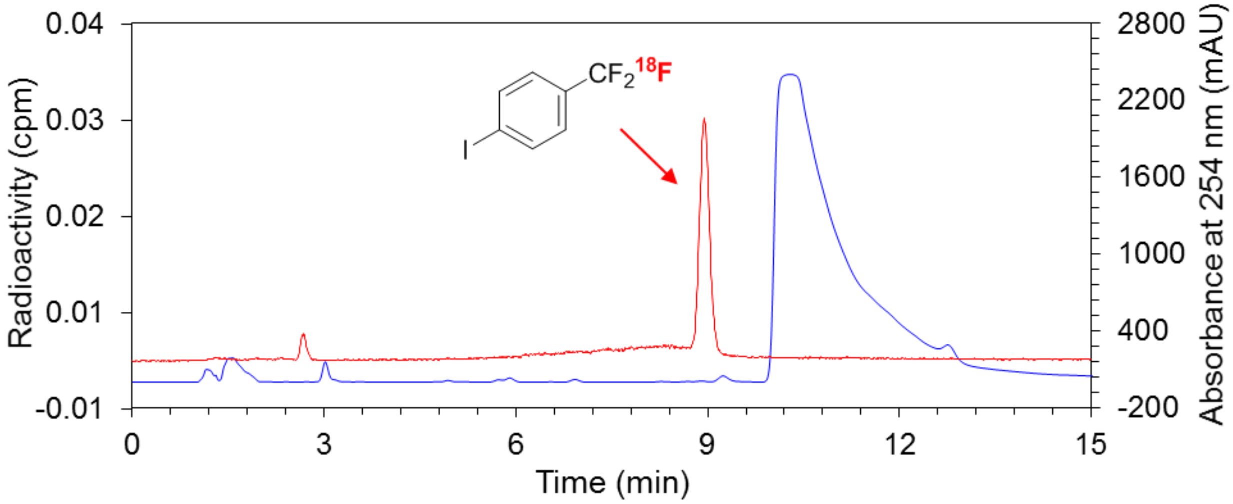

Figure S11. HPLC analysis of crude [^{18}F]1-iodo-4-(trifluoromethyl)benzene ([^{18}F]**9a**) with a mixture of 0.1% TFA water (A) and MeCN (B) as a mobile phase, with B set initally at 55% for 1 min, and then increased linarly to 80% over 10 min.

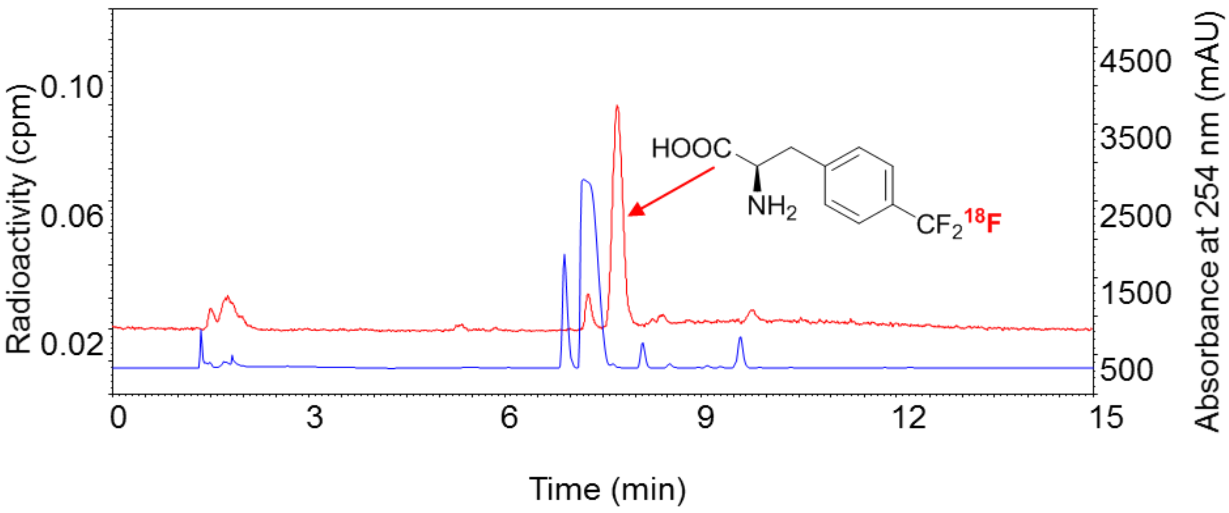

Figure S12. HPLC analysis of crude [^{18}F](*R*)-2-amino-3-(4-(trifluoromethyl)phenyl)propanoic acid ([^{18}F]**9b**) with a mixture of water (A) and MeCN (B) as mobile phase, with B set initially at 5% for 1 min, and then increased linearly to 90% over 15 min.

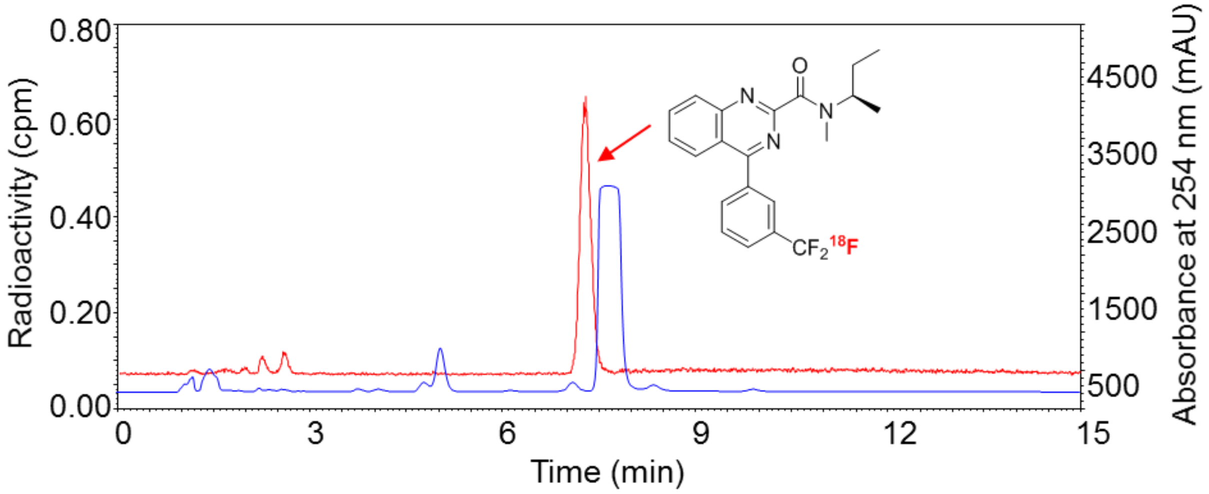

Figure S13. HPLC analysis of crude [^{18}F](*R*)-*N*-sec-butyl-*N*-methyl-4-(3-(trifluoromethyl)phenyl)quinazoline-2-carboxamide ([^{18}F]**9c**) with a mixture of 0.1% TFA water (A) and MeCN (B) as mobile phase, with B set initially at 55% for 1 min, and then increased linearly to 80% over 15 min.

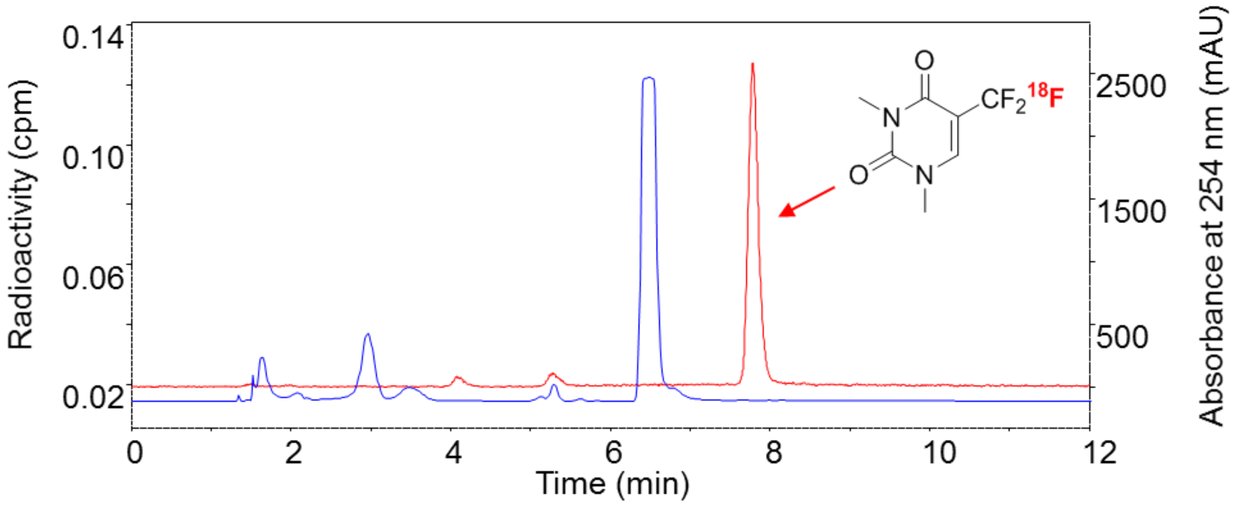

Figure S14. HPLC analysis of crude [^{18}F]1,3-dimethyl-5-(trifluoromethyl)pyrimidine-2,4(1*H*,3*H*)-dione ([^{18}F]**9d**) with a mixture of 0.1% TFA water (A) and MeCN (B) as mobile phase, with B set initially at 10% for 1 min, and then increased linearly to 80% over 15 min.

Analyses of [^{18}F]trifluoromethylarenes produced from arylboronic acids. Crude radioactive products of [^{18}F]**11c** and [^{18}F]**11d** were analyzed with a mixture of 0.1% TFA water (A) and MeCN (B) as mobile phase, with B set intially at 45% for 1 min, and then increased linearly to 80% in 15 min. All other crude radioactive products were analyzed with a mixture of 0.1% TFA water (A) and MeCN (B) as mobile phase, with B set intially at 55% for 1 min, and then increased linearly to 80% in 15 min.

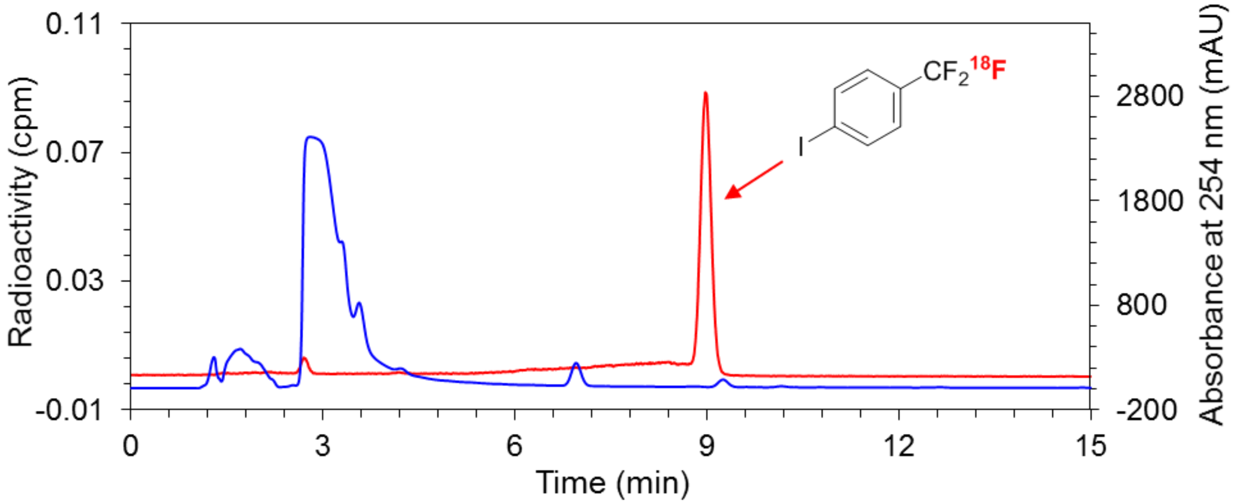

Figure S15. HPLC analysis of crude [^{18}F]4-(trifluoromethyl)iodobenzene ([^{18}F]**9a**).

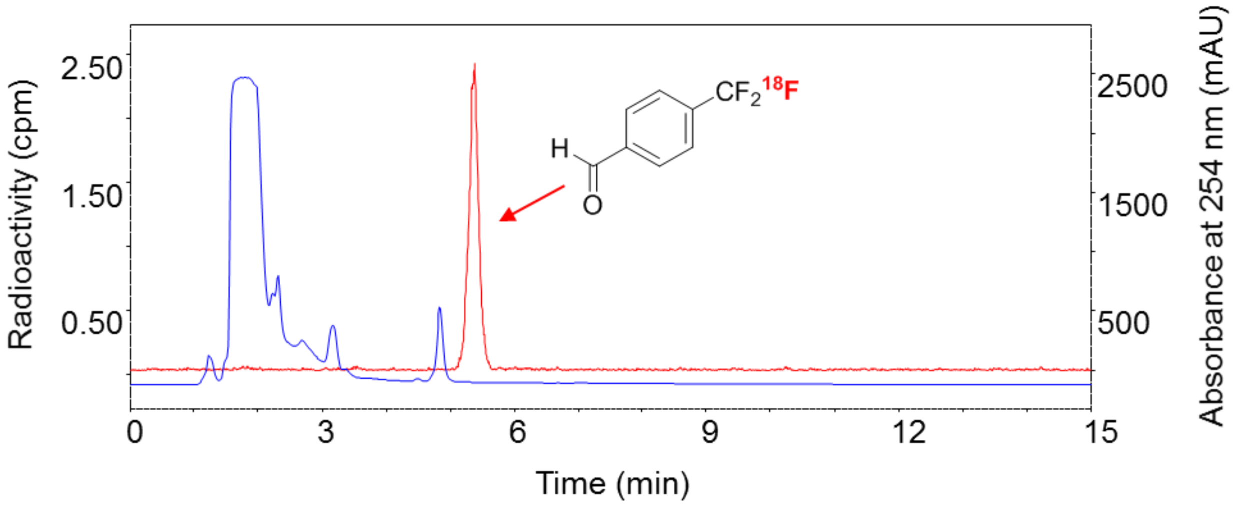

Figure S16. HPLC analysis of crude [^{18}F]4-(trifluoromethyl)benzaldehyde ([^{18}F]**11a**).

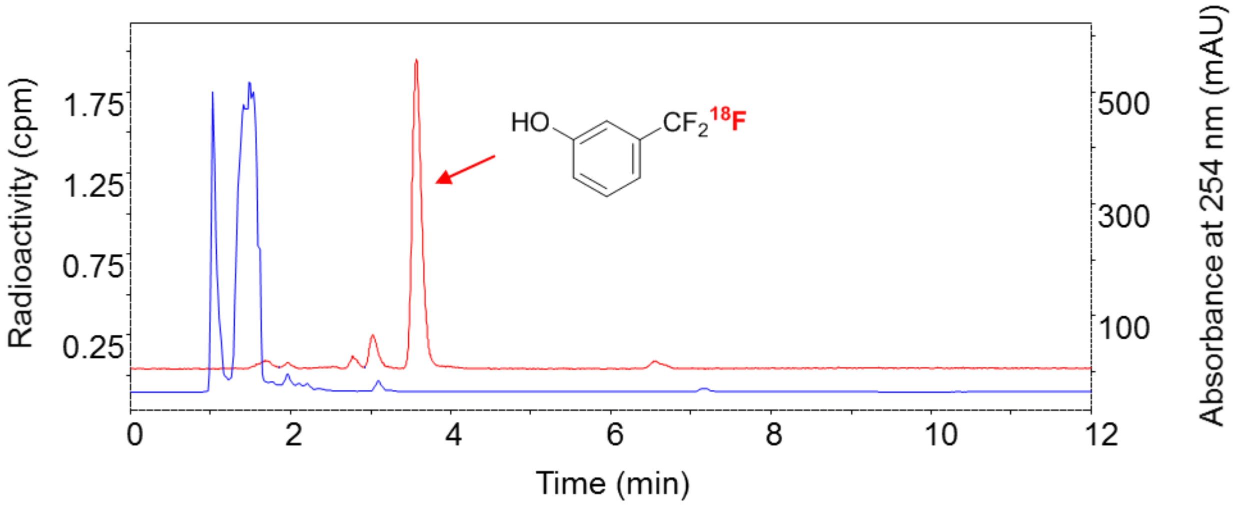

Figure S17. HPLC analysis of crude [^{18}F]3-(trifluoromethyl)phenol ([^{18}F]**11b**).

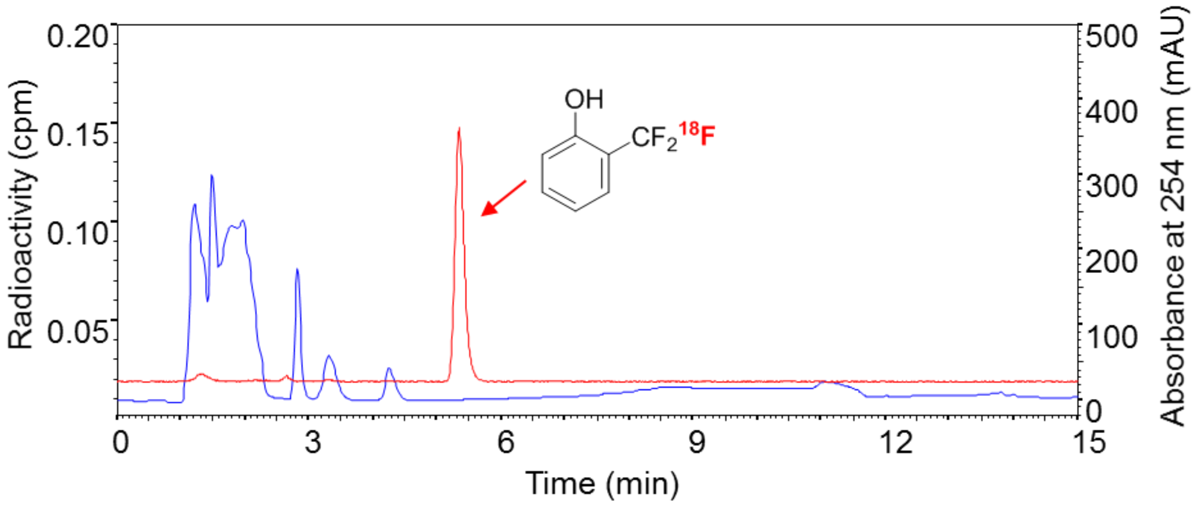

Figure S18. HPLC analysis of crude [^{18}F]2-(trifluoromethyl)phenol ([^{18}F]**11c**).

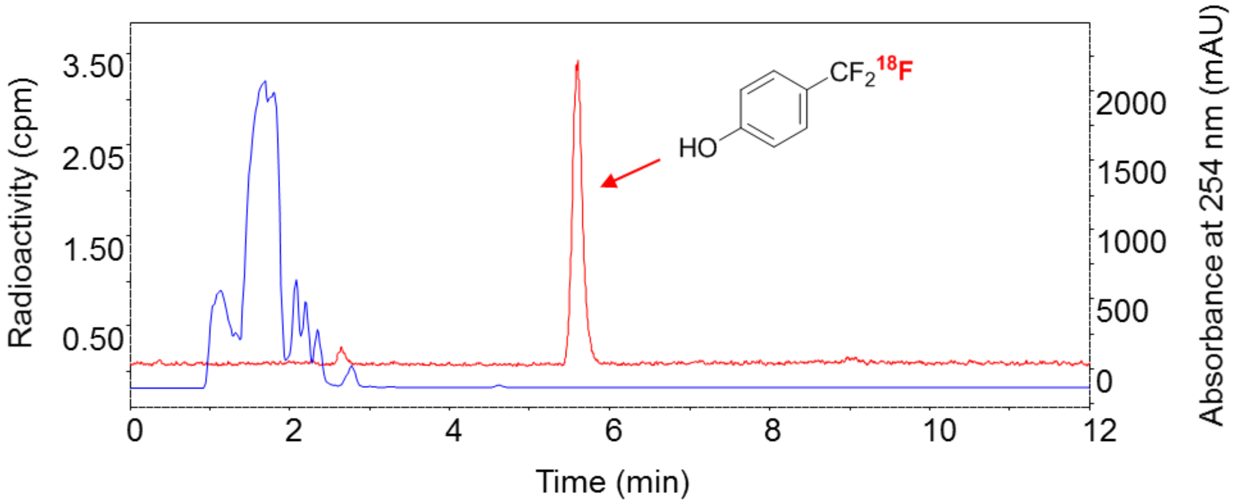

Figure S19. HPLC analysis of [^{18}F]4-(trifluoromethyl)phenol ([^{18}F]**11d**).

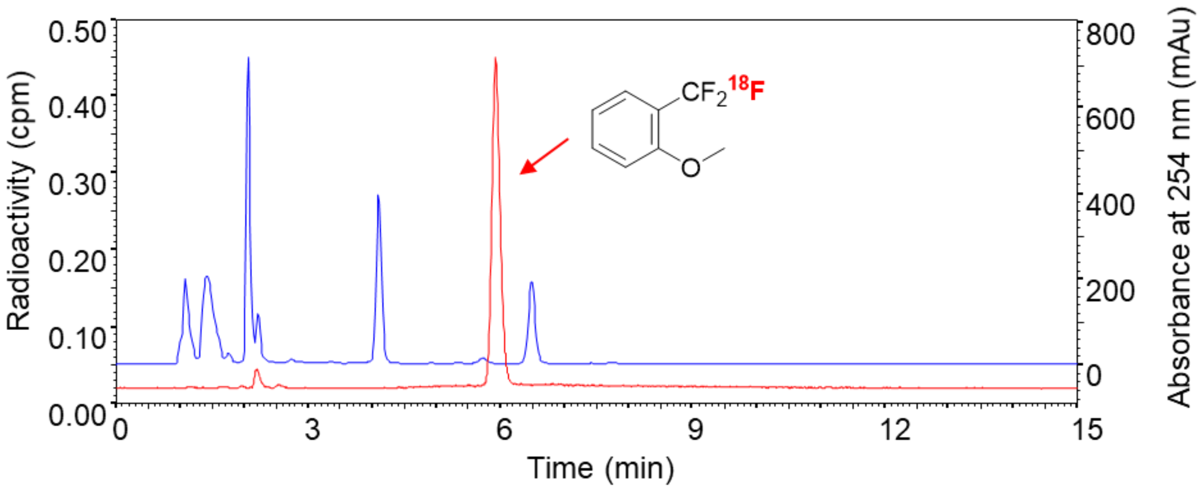

Figure S20. HPLC analysis of crude [^{18}F]1-methoxy-2-(trifluoromethyl)benzene ([^{18}F]**11e**).

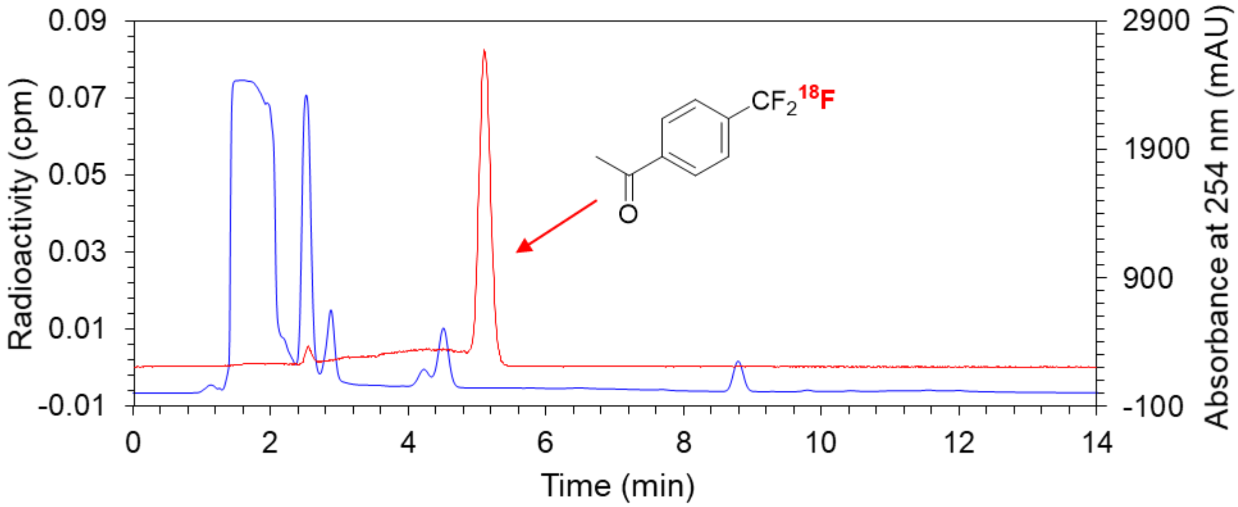

Figure S21. HPLC analysis of crude [^{18}F]1-(4-(trifluoromethyl)phenyl)ethanone ([^{18}F]**11f**).

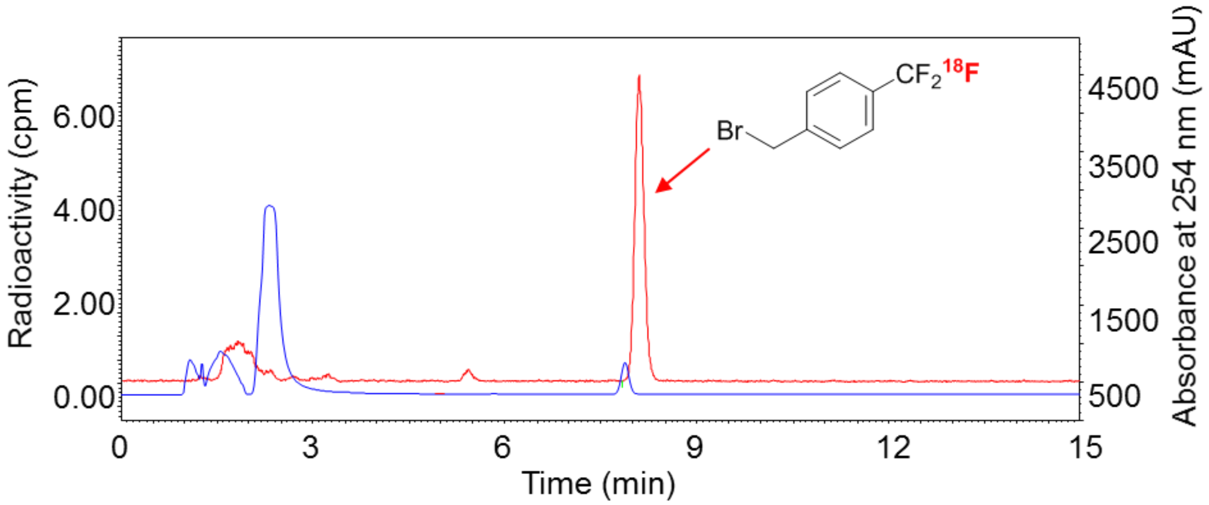

Figure S22. HPLC chromatogram for crude [^{18}F]1-(bromomethyl)-4-(trifluoromethyl)benzene ([^{18}F]**11g**).

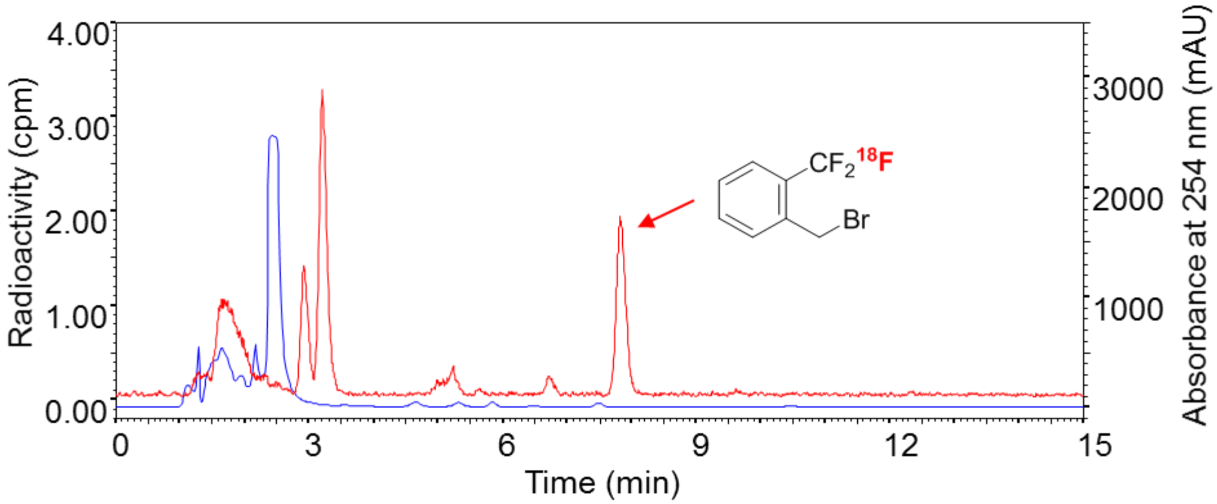

Figure S23. HPLC analysis of crude [^{18}F]1-(bromomethyl)-2-(trifluoromethyl)benzene ([^{18}F]**11h**).

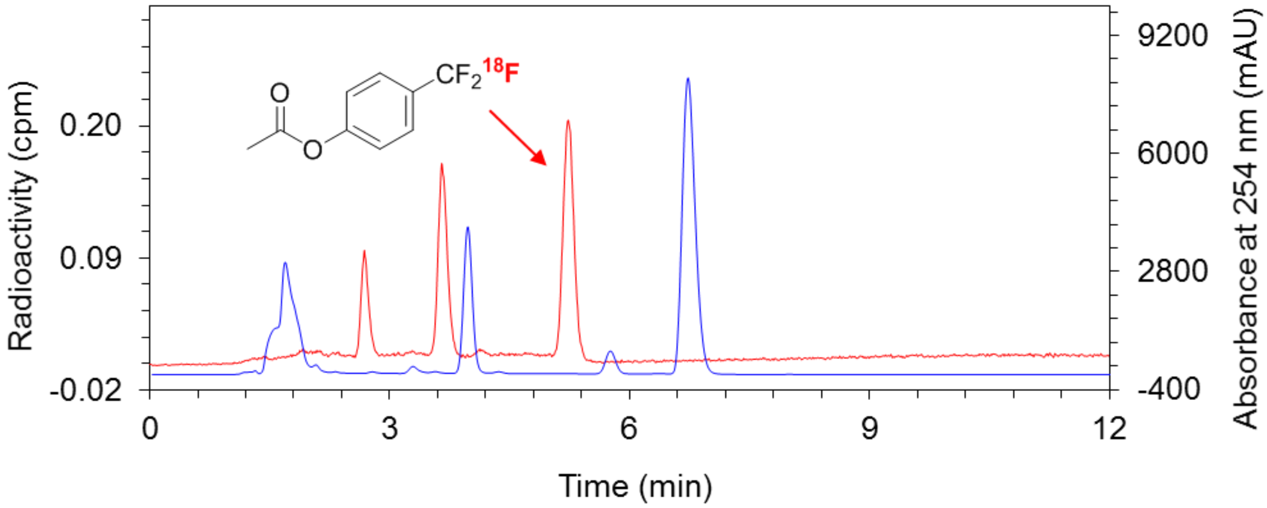

Figure S24. HPLC analysis of crude [^{18}F]4-(trifluoromethyl)phenyl acetate ([^{18}F]**11i**).

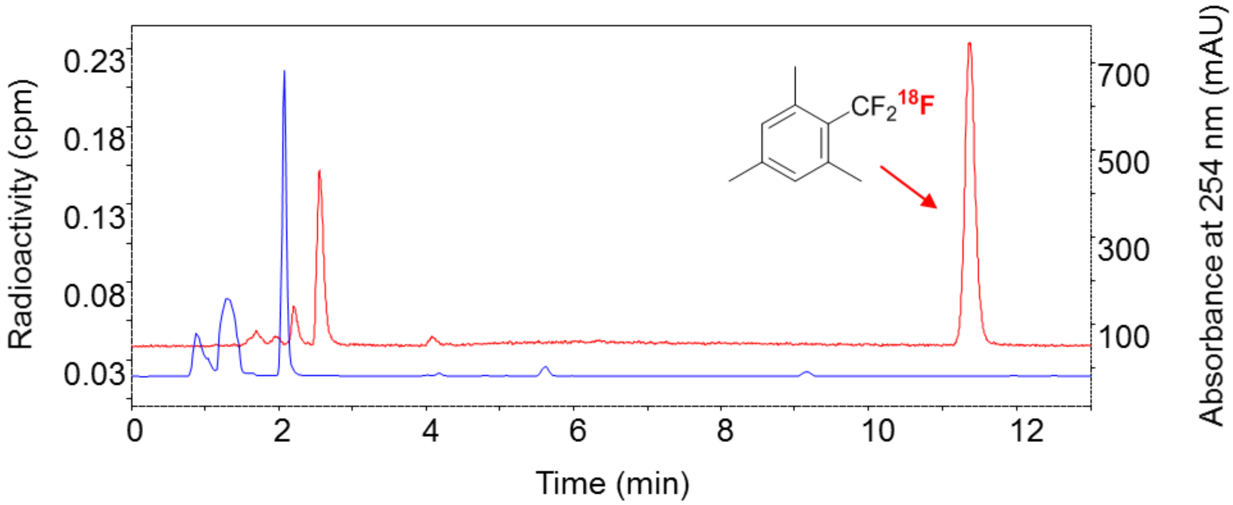

Figure S25. HPLC analysis of crude [^{18}F]1,3,5-trimethyl-2-(trifluoromethyl)benzene ([^{18}F]**11j**).

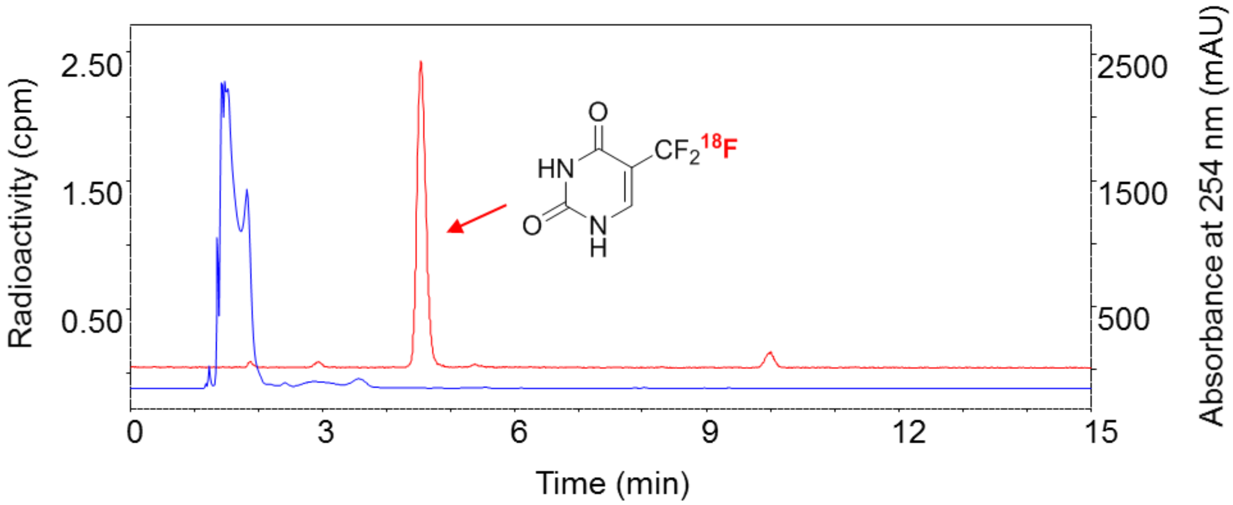

Figure S26. HPLC analysis of crude [^{18}F]5-(trifluoromethyl)pyrimidine-2,4(1*H*,3*H*)-dione ([^{18}F]**11k**).

Analyses of [^{18}F]trifluoromethylarenes produced from aryldiazonium salts. Crude radioactive products were analyzed with a mixture of 0.1% TFA water (A) and MeCN (B) as mobile phase, with B set initially at 55% for 1 min, and then increased linearly to 80% over 15 min.

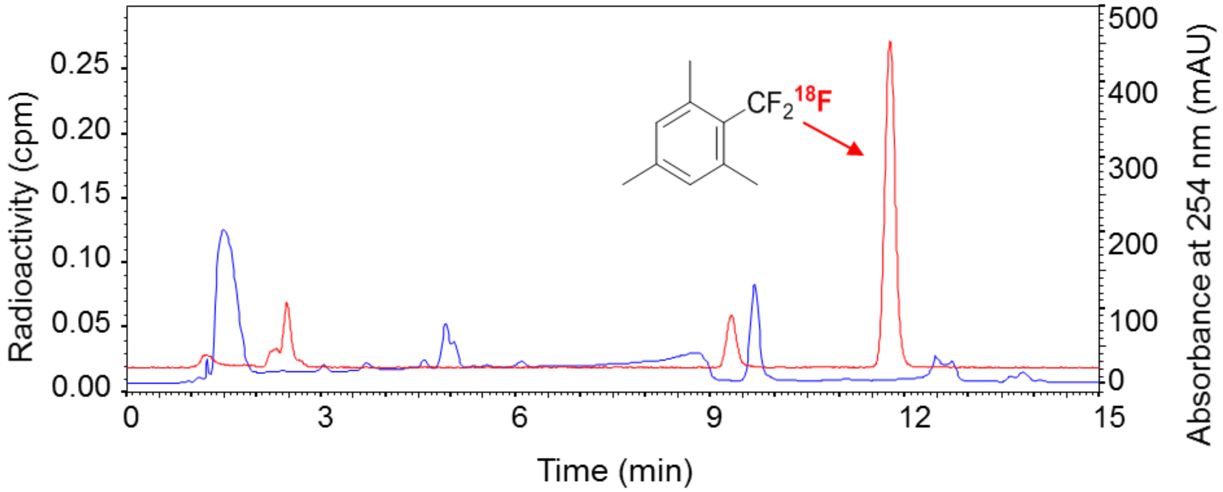

Figure S27. HPLC analysis of crude [^{18}F]1,3,5-trimethyl-2-(trifluoromethyl)benzene ([^{18}F]**11j**).

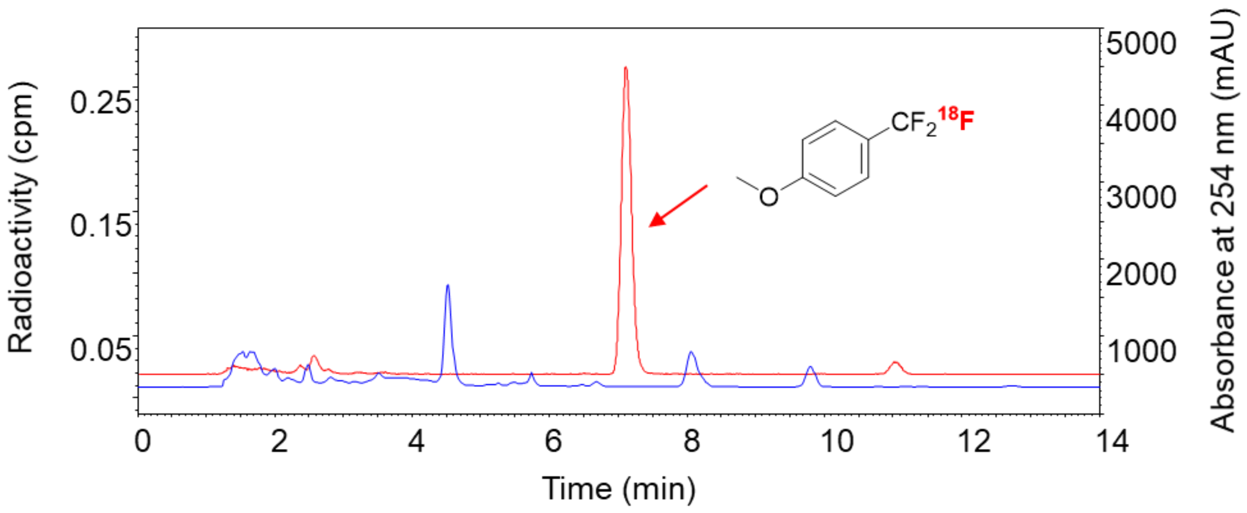

Figure S28. HPLC analysis of crude [^{18}F]1-methoxy-4-(trifluoromethyl)benzene ([^{18}F]**13a**).

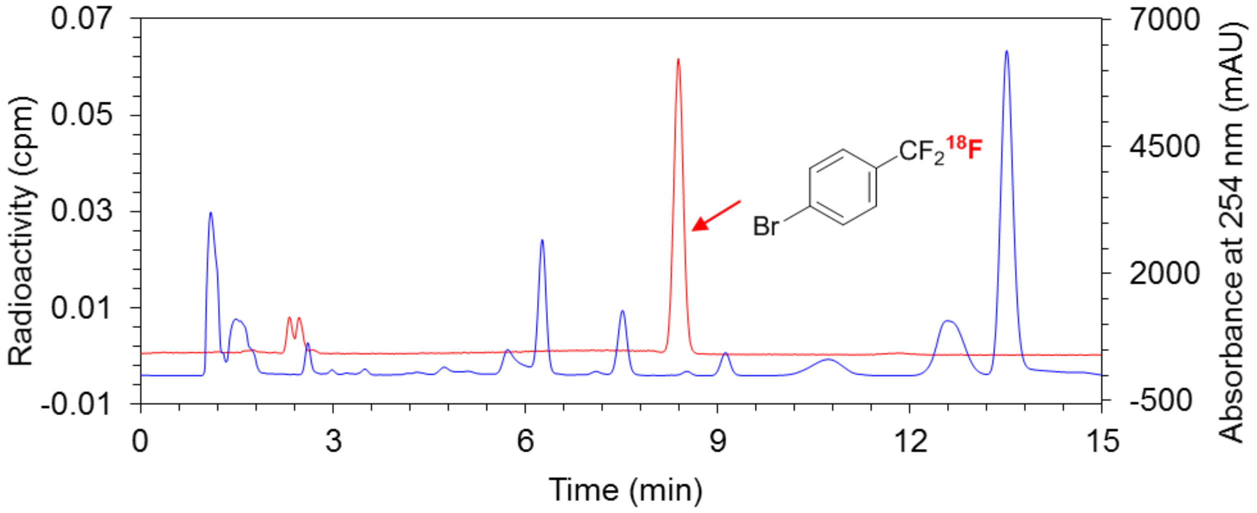

Figure S29. HPLC analysis of crude [^{18}F]1-bromo-4-(trifluoromethyl)benzene ([^{18}F]**13b**).

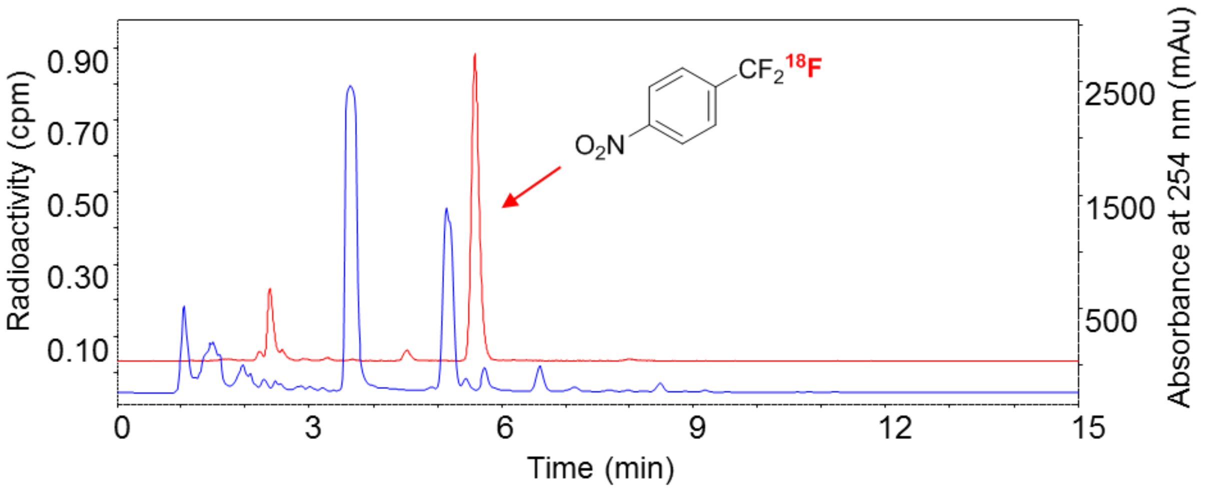

Figure S30. HPLC analysis of crude [^{18}F]1-nitro-4-(trifluoromethyl)benzene ([^{18}F]**13c**).

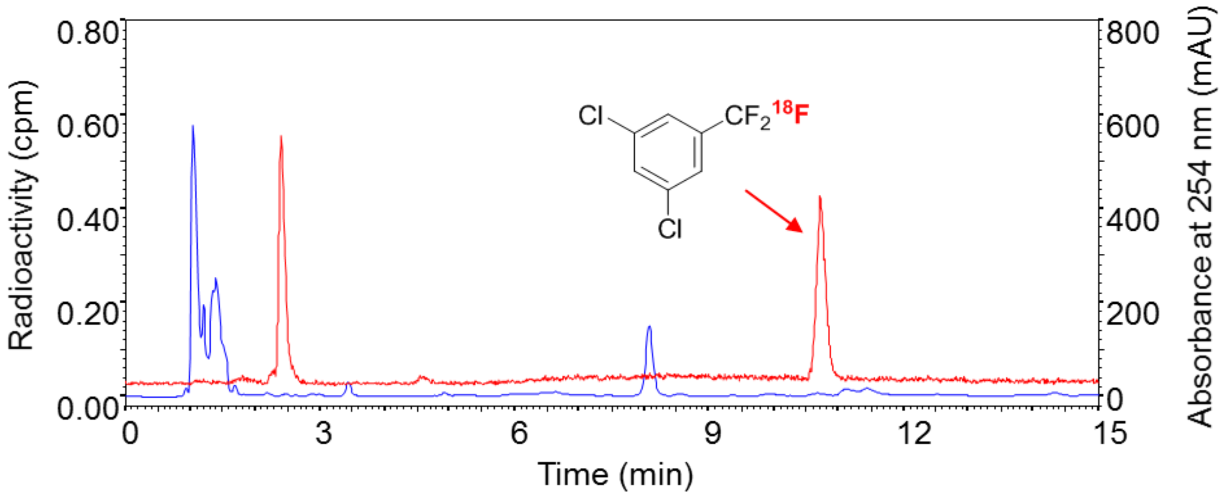

Figure S31. HPLC analysis of crude [^{18}F]1,3-dichloro-5-(trifluoromethyl)benzene ([^{18}F]**13d**).

Determination of Molar Activity (A_m) of [^{18}F]5

The molar actiivty of [^{18}F]**5** was determined to provide estimates of the molar activity of [^{18}F]fluoroform used in its synthesis. Set amounts of reference α-(trifluoromethyl)benzhydrol (**5**) (up to 1 ng/µL) were injected onto a Luna C18 column (10 µm, 250 × 4.6 mm) eluted at 2 mL/min with a mixture of water (A) and MeCN (B) as mobile phase, with B set initially at 45% for 1 min, and then increased to 80% over 20 min. Absorbance response (peak area) at 215 nm was plotted versus the mass of **5** injectate to provide a standard curve (**Fig. S32**). The mass of **5** (*x* µmol) associated with an injectate of [^{18}F]**5** (100 µL) was then measured with this HPLC method. The radioactivity correspondingly eluted from the column was measured with a calibrated ionization detector and decay-corrected to the value (y Bq) at the end of radionuclide production. The molar activity A_m value was calculated as *y/x* Bq/µmol (*n* = 10).

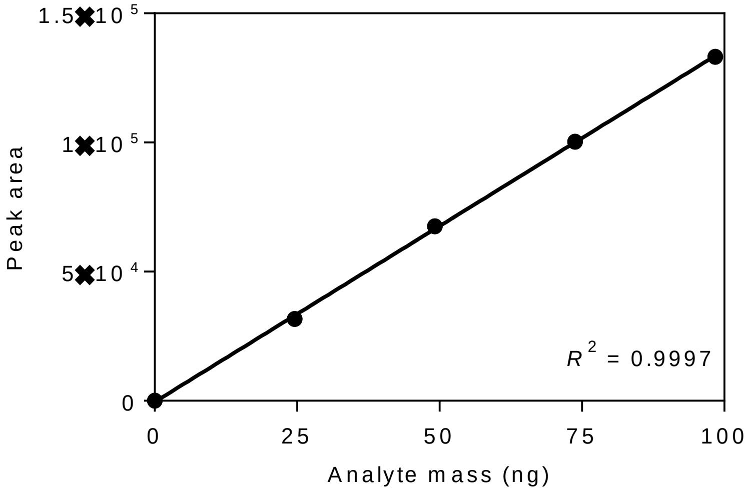

Figure S32. A standard curve for HPLC measurement of α-(trifluoromethyl)benzhydrol (**5**).

Comparison of the Molar Activity of [^{18}F]5 with that of a Radiotracer, [^{18}F]OGA-1, Produced by *S*NAr from the Same Source of [^{18}F]Fluoride Ion

On several days, a single batch of cyclotron-produced [^{18}F]fluoride ion was used to produce [^{18}F]**OGA-1** (an *O*-GlcNAcase imaging agent) according to a conventional *S*NAr reaction (Fig. S33),[2] and to produce [^{18}F]**5**. Both [^{18}F]**OGA-1** and [^{18}F]**5** were measured for molar radioactivity, corrected to the end of radionuclide production.

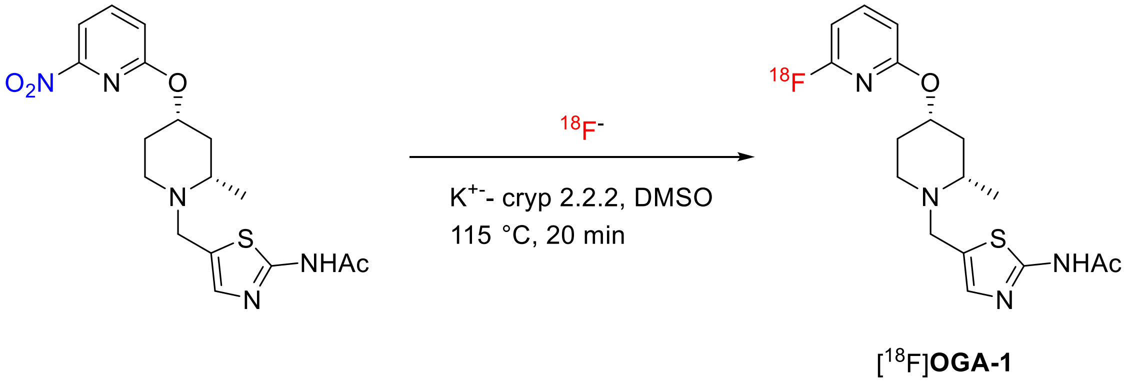

Figure S33. Synthesis of [^{18}F]**OGA-1**.[2]

Comparison of the Molar Activity of [^{18}F]5 with that of [^{18}F]9a Produced from the Same Source of [^{18}F]Fluoride Ion

A single batch of cyclotron-produced [^{18}F]fluoride ion was used to produce [^{18}F]**5** and [^{18}F]**9a** ([^{18}F]1-iodo-4-(trifluoromethyl)benzene) to test whether the presence of added HF in the radiosynthesis resulted in

dilution of molar activity. No significant difference was found between the molar activities of these labeled products (P = 0.79) (n = 2).

Calculation of Dilution of A_m (for Fig. 5A)

The dilution of molar activity (A_m) occurring during the conversion of [^{18}F]fluoromethane into [^{18}F]fluoroform was calculated as:

$$(A_m \text{ of } [^{18}\text{F}]\textbf{OGA-1})/(A_m \text{ of } [^{18}\text{F}]\textbf{5})$$

where each A_m value is decay-corrected to the same time point (the end of radionuclide production).

Table S1. Calculation of A_m dilution during the conversion of [^{18}F]fluoromethane into [^{18}F]fluoroform before use of GC purification.

Entry	A_m Dilution before use of GC purification
1	5.04
2	6.66
3	6.94
4	9.22
5	11.3
6	9.36
Mean ± SD	8.1 ± 2.28

Table S2. Calculation of A_m dilution during the conversion of [^{18}F]fluoromethane into [^{18}F]fluoroform after use of GC purification.

Entry	A_m Dilution after use of GC purification
1	0.64
2	3.38
3	4.57
4	2.51
Mean ± SD	2.78 ± 1.65

The means ± SDs in Table S1 and Table S2 by T-test are significantly different (P = 0.003).

Calculation of Carrier Dilutions (for Fig. 5B)

The dilution of carrier occurring during the conversion of [^{18}F]fluoromethane to [^{18}F]fluoroform was calculated as: $\mu mol_{out}/\mu mol_{in}$

where μmol_{out} = [(radioactivity of [^{18}F]fluoroform (GBq) leaving the CoF_3 column)/(A_m of [^{18}F]**5** (GBq/µmol)]

and μmol_{in} = [(radioactivity (GBq) of [^{18}F]fluoromethane entering CoF_3 column)/(A_m of [^{18}F]**OGA-1** (GBq/µmol)]

Each radioactivity and A_m value were decay-corrected to the same time point.

Table S3. Estimates of $\mu mol_{out}/\mu mol_{in}$ before use of GC purification

Entry	y_1 (GBq_{in})	A_m of [^{18}F]OGA-1 (GBq/µmol)	μmol_{in}	w_1 (GBq_{out})	A_m of [^{18}F]5 (GBq/µmol)	μmol_{out}	$\mu mol_{out}/\mu mol_{in}$
1	2.67	91.3	0.029	0.80	18.1	0.044	1.50
2	3.82	168	0.023	0.16	25.2	0.006	0.28
3	2.90	157	0.018	0.86	22.6	0.005	0.26

4	3.93	131	0.030	0.59	14.2	0.011	0.35
5	4.74	179	0.027	0.49	15.8	0.031	1.17
6	4.65	86.9	0.054	1.49	9.29	0.161	3.00
Mean ± SD							1.10 ± 1.07

y_1 = [^{18}F]fluoromethane (GBq) entering CoF_3 column
µmol$_{in}$ = y_1/A_m of [^{18}F]**OGA-1**
w_1 = [^{18}F]fluoroform (GBq) leaving the CoF_3 column
µmol$_{out}$ = w_1/A_m of [^{18}F]**5**

Table S4. Estimates of µmol$_{out}$/µmol$_{in}$ after use of GC purification

**Entry**	***y*$_2$ (GBq$_{in}$)**	***A*$_m$ of [^{18}F]OGA-1 (GBq/µmol)**	**µmol$_{in}$**	***w*$_2$ (GBq$_{out}$)**	***A*$_m$ of [^{18}F]5 (GBq/µmol)**	**µmol$_{out}$**	**µmol$_{out}$/µmol$_{in}$**
1	11.5	104	0.110	2.84	163	0.017	0.16
2	19.0	336	0.057	4.33	99.4	0.044	0.77
3	6.31	144	0.044	1.58	31.6	0.050	1.15
4	5.74	103	0.056	1.54	41.0	0.038	0.67
Mean ± SD							0.69 ± 0.41

Y_2 = [^{18}F]fluoromethane (GBq) entering CoF_3 column
µmol$_{in}$ = y_1/A_m of [^{18}F]**OGA-1**
w_2 = [^{18}F]fluoroform (GBq) leaving the CoF_3 column
µmol$_{out}$ = w_1/A_m of [^{18}F]**5**
The means ± SDs in Table S3 and Table S4 by T-test are significantly different (*P* = 0.43).

References

1 Doyle, M. P. & Bryker, W. J. Alkyl nitrite-metal halide deamination reactions. 6. Direct synthesis of arenediazonium tetrafluoroborate salts from aromatic amines, tert-butyl nitrite, and boron trifluoride etherate in anhydrous media. *J. Org. Chem.* **44**, 1572-1574 (1979).

2 Paul, S. *et al.* Evaluation of a PET radioligand to image *O*-GlcNAcase in brain and periphery of rhesus monkey and knock-out mouse. *J. Nucl. Med.* **60**, 129-134 (2019).
